# A Small Cysteine-Rich Protein from the Asian Soybean Rust Fungus, *Phakopsora pachyrhizi*, Suppresses Plant Immunity

**DOI:** 10.1371/journal.ppat.1005827

**Published:** 2016-09-27

**Authors:** Mingsheng Qi, Tobias I. Link, Manuel Müller, Daniela Hirschburger, Ramesh N. Pudake, Kerry F. Pedley, Edward Braun, Ralf T. Voegele, Thomas J. Baum, Steven A. Whitham

**Affiliations:** 1 Department of Plant Pathology and Microbiology, Iowa State University, Ames, Iowa, United States of America; 2 Institut für Phytomedizin, Universität Hohenheim, Stuttgart, Germany; 3 Amity Institute of Nanotechnology, Amity University Uttar Pradesh, Noida, India; 4 Foreign Disease-Weed Science Research Unit, United States Department of Agriculture–Agricultural Research Service, Ft. Detrick, Maryland, United States of America; CSIRO, AUSTRALIA

## Abstract

The Asian soybean rust fungus, *Phakopsora pachyrhizi*, is an obligate biotrophic pathogen causing severe soybean disease epidemics. Molecular mechanisms by which *P*. *pachyrhizi* and other rust fungi interact with their host plants are poorly understood. The genomes of all rust fungi encode many small, secreted cysteine-rich proteins (SSCRP). While these proteins are thought to function within the host, their roles are completely unknown. Here, we present the characterization of *P*. *pachyrhizi* effector candidate 23 (*Pp*EC23), a SSCRP that we show to suppress plant immunity. Furthermore, we show that *Pp*EC23 interacts with soybean transcription factor *Gm*SPL12l and that soybean plants in which *GmSPL12l* is silenced have constitutively active immunity, thereby identifying *Gm*SPL12l as a negative regulator of soybean defenses. Collectively, our data present evidence for a virulence function of a rust SSCRP and suggest that *Pp*EC23 is able to suppress soybean immune responses and physically interact with soybean transcription factor *Gm*SPL12l, a negative immune regulator.

## Author Summary

Asian soybean rust is a disease caused by the rust fungus, *Phakopsora pachyrhizi*, which was first described in Japan in 1902 and eventually reached North and South America in the early 2000s. Like other rust fungi, *P*. *pachyrhizi*, is capable of causing disease epidemics that severely reduce crop yields. Rust fungi require living host tissue in order to complete their life cycles, and thus, we expect that they produce and secrete effector proteins that are transferred into plant cells to interfere with immune responses. Small proteins that are rich in the amino acid cysteine and predicted to be secreted have been proposed to be likely candidates for effector proteins. Here, we report that a secreted, small cysteine-rich protein from *P*. *pachyrhizi* suppresses plant immunity, and furthermore, it interacts with a soybean transcription factor protein. Interestingly, silencing of this transcription factor results in plants that have increased expression of defense marker genes and enhanced disease resistance, which demonstrates that it is a negative regulator of soybean immune responses. Our work shows that, indeed, a small cysteine-rich protein from a rust fungus can suppress plant immunity, and furthermore, it identifies a new function for SQUAMOSA PROMOTER BINDING-LIKE (SPL) transcription factors as components of the defense regulatory networks that holds immune responses in check.

## Introduction

Rust fungi comprise many pathogenic species in the order Pucciniales that cause devastating plant diseases [1–3]. They are notable for their obligate biotrophy and complex life cycles that include distinct spore-producing stages and host specialization. As rust fungi colonize their hosts, they form specialized hyphal structures, called haustoria, within their host plant cells, and these structures are critical for nutrient uptake, metabolic processes, and delivery of effector proteins that manipulate host immune systems [4,5]. Many rust candidate effector proteins have been predicted through whole-genome sequencing and *in planta* expression studies [3,6–8].

The most well-studied effector proteins are from four different rust species: AvrM, AvrL567, AvrP123, and AvrP4 from *Melampsora lini* (flax rust), Rust Transferred Protein (RTP1) from *Uromyces fabae* (broad bean rust), PGTAUSPE10-1 from *Puccinia graminis* f. sp. *tritici* (wheat stem rust), and most recently, PEC6 from *Puccinia striiformis* f. sp. *tritici* (wheat stripe rust). AvrM, AvrL567, AvrP123, AvrP4 and PGTAUSPE10-1 have been studied intensely for their avirulence properties defined by their abilities to trigger R-protein mediated immune responses [9–12]. RTP1 was the first rust protein proven to be directly transferred from haustoria to plant cells during infection [11], and moreover, it was shown to form fibrils and be a protease inhibitor [9], although its targets and detailed mechanisms still remain unclear. PEC6 suppresses basal defense responses in non-host and host plants and interacts with adenosine kinases [13]. Pathogen-free assays further demonstrate the autonomous entry of AvrP4, AvrM and AvrL567 into plant cells [14–16]. Additionally, AvrP4, AvrP123, and PEC6 all are small, secreted cysteine-rich proteins (SSCRP) [13,14].

Asian soybean rust (ASR), caused by *Phakopsora pachyrhizi*, is a serious threat to soybean production. The rapid lifecycle and the production of large numbers of infectious urediospores under optimal environmental conditions lead to high inoculum levels and potentially high yield losses [17]. To date, six *P*. *pachyrhizi R* genes have been identified [18], but monogenic resistance to ASR is rapidly overcome by virulent *P*. *pachyrhizi* isolates [19]. The variation in *P*. *pachyrhizi* virulence makes research on conserved effectors highly significant, because they may provide new targets for broad spectrum resistance to many virulent isolates of this pathogen as well as provide new insights into mechanisms by which rust pathogens manipulate host immune systems. Recently, haustoria of *P*. *pachyrhizi* were purified and the haustorial transcriptome was sequenced and analyzed, which provided a resource for predicting secreted *P*. *pachyrhizi* effector candidates (*Pp*ECs) [8]. Among the *Pp*ECs were several SSCRP, *i*.*e*., a class of proteins that had been hypothesized to be strong candidates for rust fungus effectors [20].

Suppression of soybean immunity is an expected function for some *Pp*ECs that promote *P*. *pachyrhizi* virulence. This is necessary because plant immune recognition systems activate defenses in response to conserved features or specific effector proteins produced by pathogens [21,22]. Pathogen-associated molecular patterns (PAMPs) are conserved features of pathogens that can elicit a type of basal defense known as PAMP-triggered immunity (PTI) when they are perceived by pattern recognition receptors (PRR) on the plant cell surface [23–25]. Basal defense responses include the deposition of callose in the cell walls, a reactive oxygen species (ROS) burst, and transcriptional up-regulation of immune-related genes, all of which restrict pathogen proliferation [26]. However, these defense responses are suppressed by successful pathogens through multiple means including secretion of effector proteins [22]. In return, host plants have evolved the more specific and much stronger effector-triggered immune (ETI) responses, such as the hypersensitive response (HR), which is a form of programmed cell death. HR is often mediated by nucleotide binding site leucine-rich repeat (NBS-LRR) proteins that recognize the presence of specific avirulence (former effector) proteins [27]. Even though basal defense and HR can be roughly discriminated based on the response strength and specificity, they share many signaling components [28].

We are interested in identifying *Pp*ECs that suppress immunity and characterizing the mechanisms by which they interfere with soybean immune signaling. To initiate this work, we performed functional screens designed to identify *Pp*ECs that suppress defense responses elicited by a bacterial pathogen, *Pseudomonas syringae* pv. *tomato* (*Pst*). One SSCRP, which we named *Pp*EC23, was unique in its ability to suppress HR and basal defense responses. The major feature of *Pp*EC23 is a tandem repeat of a ten-cysteine motif that appears in the predicted secretomes of other rust fungi [1,3,8], and it interacts with soybean transcription factor *Gm*SPL12l (SQUAMOSA promoter-binding-like protein 12 like). RNA silencing of *GmSPL12l* indicates that this transcription factor functions as a negative regulator of soybean defense responses. While prior work documented *avirulence* functions for a few SSCRPs, *i*.*e*. their detection by the plant immune system leading to plant defenses, the current study establishes that SSCRP *Pp*EC23 has functions consistent with a virulence factor that regulates host plant immunity.

## Results

### *Pp*EC23 suppresses HR induced by *Pst* strain DC3000 in soybean, *Nicotiana benthamiana*, and tobacco

**Fig 1:**
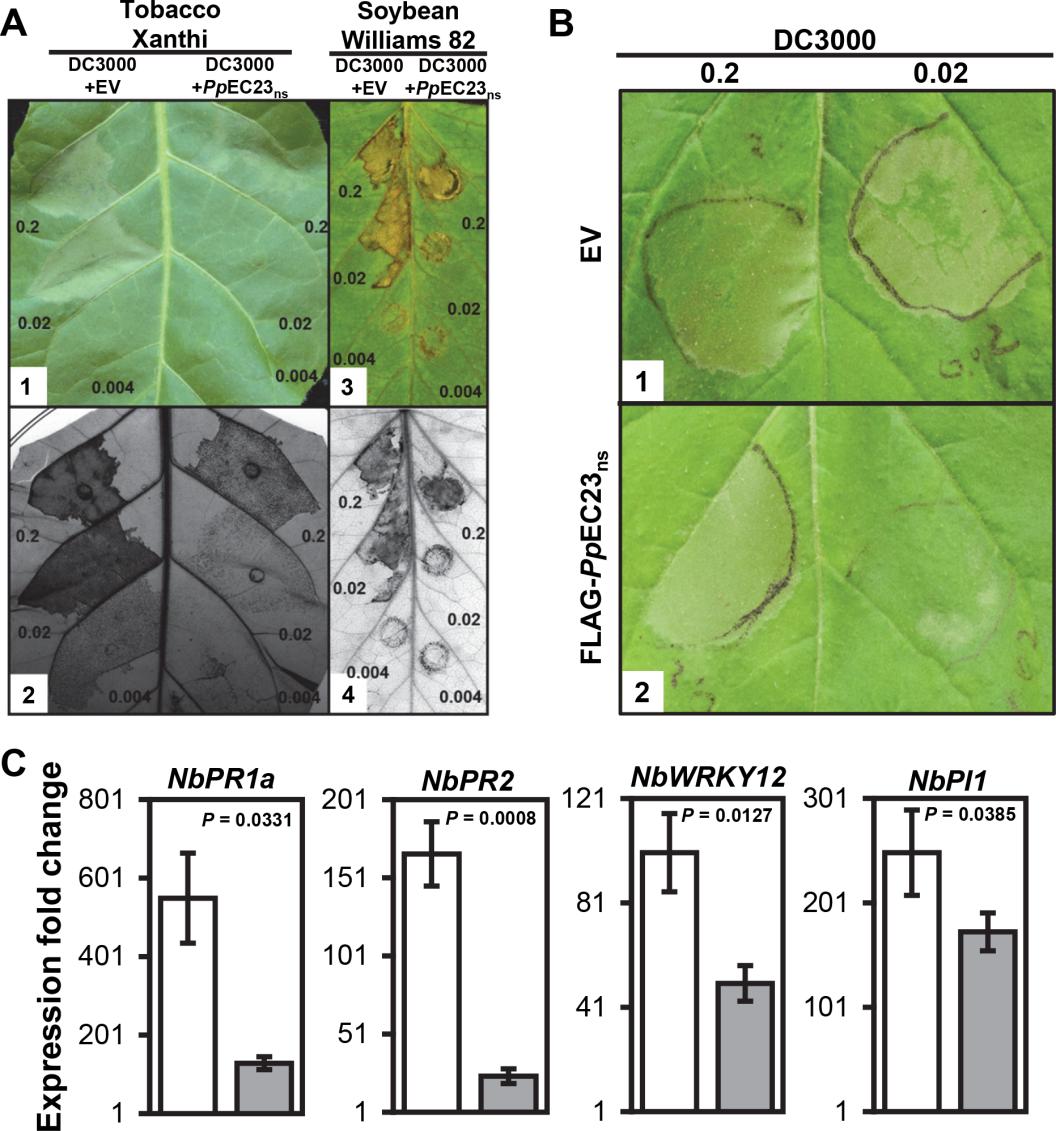
*Pp*EC23 suppresses HR induced by *Pst* DC3000. A. HR on leaves of *N*. *tabacum* cv. Xanthi (A1, A2) and *G*. *max* cv. Williams 82 (A3, A4) infiltrated with *Pst* DC3000 carrying empty pEDV6 vector (EV) (left half) and *Pst* DC3000 carrying pEDV6::*Pp*EC23_ns_ (right half). Leaves were infiltrated with bacteria at OD _600 nm_ = 0.2, 0.02, and 0.004 (approximately equal to 10^8^, 10^7^ and 2x10^6^ CFU (colony forming units) / mL, respectively [32]). Leaves were photographed at 18 h post infiltration (hpi) for tobacco (A1) and at 30 hpi for soybean (A3) and then stained with trypan blue to detect non-viable cells (A2, A4). B. HR on leaves of *N*. *benthamiana* stably transformed with EV (B1) and FLAG-*Pp*EC23_ns_ (B2) infiltrated with *Pst* DC3000. Leaves were infiltrated with bacteria at OD _600 nm_ = 0.2 (left), 0.02 (right). Leaves were photographed at 18 hpi. Representative images are shown (n ≥ 8). C. The expression fold change of immune marker genes *PR1a*, *PR2*, *WRKY12*, and *PI1* in the leaves of *N*. *benthamiana* stably transformed with EV (white bars) and FLAG-*Pp*EC23_ns_ (gray bars) at 16 hpi with *Pst* DC3000. *NbAct1* was used as the internal reference gene. A *t*-test was performed for each pair-wise comparison and the *P* value for each comparison is shown. Four biological and four technical replicates were performed.

We wanted to determine if a *Pp*EC could suppress plant immune responses. To test this, we cloned 82 of 156 *Pp*ECs identified by Link et al. [8] minus their predicted signal peptides (*Pp*EC_ns_) into the bacterial type III secretion system (T3SS) vector, pEDV6 [29]. The pEDV6::*Pp*EC_ns_ constructs plus the empty pEDV6 were introduced into *Pseudomonas syringae* pv. *tomato* strain DC3000 (*Pst* DC3000), which elicits an HR on our experimental plants *N*. *benthamiana*, tobacco (*N*. *tabacum* cv. Xanthi), and soybean (*Glycine max* cv. Williams 82) [30,31]. As expected, infiltration of *N*. *benthamiana*, *N*. *tabacum*, and soybean leaves with *Pst* DC3000 carrying the empty vector (EV) at optical densities (OD) _600 nm_ of 0.2 and 0.02 caused strong macroscopic HR (Fig 1A1 and 1A3, S1A and S1B Fig, left side of leaves). Of the 82 *Pp*EC_ns_ screened for suppression of *Pst* DC3000-induced HR, pEDV6::*Pp*EC23_ns_ was unique in its ability to delay and reduce the HR (Fig 1A1 and 1A3, S1A and S1B Fig, right side of leaves). Infiltrated areas on soybean leaves are much smaller compared to those on *Nicotiana* leaves, and more force is required to introduce the inoculum, so there is an obvious wound at the infiltration site. Therefore, we only kept soybean leaves in which the inoculum had clearly expanded beyond the infiltration site and photographed them two days later. To confirm this HR suppression phenotype, trypan blue staining was performed to detect dead tobacco and soybean cells (Fig 1A2 and 1A4). For both tobacco and soybean, trypan blue staining was reduced in leaf areas inoculated with *Pst* DC3000 expressing *Pp*EC23_ns_ at each inoculum density compared to *Pst* DC3000 carrying empty pEDV6, which confirmed the phenotype of weakened HR.

To exclude the possibility that the expression of *Pp*EC23_ns_ had a negative effect on the general viability of *Pst* DC3000, a bacterial growth assay was performed. The growth curves for *Pst* DC3000 carrying *Pp*EC23_ns_ and *Pst* DC3000 carrying the empty vector were similar, indicating that the delayed and reduced HR was not due to the reduced viability in Hrp-inducing minimal medium (S2 Fig). To further investigate whether the reduced HR was a direct effect of expressing *Pp*EC23 in *Pst* DC3000, we tested if pEDV6::*Pp*EC23_ns_ could suppress *Pst* DC3000-induced HR when expressed *in trans* from the non-pathogenic *P*. *fluorescens* strain EtHAn [33]. EtHAn carrying *Pp*EC23_ns_ was diluted to an OD _600 nm_ of 0.2 and then co-inoculated with serially diluted *Pst* DC3000 on *N*. *benthamiana* (S1C–S1F Fig). At 42 hpi, EtHAn expressing *Pp*EC23_ns_ had clearly suppressed HR caused by *Pst* DC3000 at an OD _600nm_ of 0.02, which was in contrast to EtHAn carrying empty pEDV6 that did not suppress the HR (S1E and S1F Fig). These data support the conclusion that the ability of *Pp*EC23 to suppress HR was not due to general effects on *Pst* DC3000, and they further indicate that HR suppression was the result of *Pp*EC23’s function *in planta*.

To directly test if *Pp*EC23 suppresses HR *in planta*, stably transformed *N*. *benthamiana* plants expressing a FLAG-*Pp*EC23_ns_ fusion protein under control of the *Cauliflower mosaic virus* 35S promoter were generated. The accumulation of FLAG-*Pp*EC23_ns_ protein was confirmed in eight independent transgenic lines by Western blot using an anti-FLAG antibody (S3 Fig). We serially diluted *Pst* DC3000 and inoculated plants from four independent transgenic lines with the highest expression of FLAG-*Pp*EC23_ns_, along with empty vector transformed controls. On control plants, *Pst* DC3000 caused strong HR when inoculated at an OD _600 nm_ of 0.02 and 0.2 (Fig 1B1). However, on FLAG-*Pp*EC23_ns_ transformants, HR was clearly suppressed in response to *Pst* DC3000 at an OD _600 nm_ of 0.02 (Fig 1B2). These results demonstrate that *Pp*EC23 suppresses *Pst* DC3000-induced ETI through an activity *in planta*.

We hypothesized that suppression of HR would be accompanied by decreased mRNA accumulation of plant immune marker genes. To test this, quantitative reverse transcriptase-PCR (qRT-PCR) was performed to detect whether *Pp*EC23 had an effect on the expression of four genes that were previously shown to represent immune responses in *Nicotiana* species: pathogenesis related genes *PR1a* and *PR2* [34], *WRKY12* transcription factor [35,36], and protease inhibitor 1 (*PI1*) [34,37,38]. *N*. *benthamiana* plants transformed with FLAG-*Pp*EC23_ns_ or empty vector were inoculated with *Pst* DC3000 at an OD _600 nm_ of 0.02 and samples were collected 16 hpi, which was prior to the appearance of macroscopic cell death. The expression of all four genes was induced in control and FLAG-*Pp*EC23_ns_ plants in response to *Pst* DC3000, but the levels of induction were much less in FLAG-*Pp*EC23_ns_ plants compared with plants transformed with the empty vector (Fig 1C). The lower expression was significant for all four genes, especially for *PR1a* and *PR2*, with 4.3 and 7 fold lower fold changes, respectively. These data show that *Pp*EC23 suppresses plant immune responses and it dampens accompanying downstream transcriptional activation.

### *PpEC23* encodes a modular SSCRP that is present in diverse *P*. *pachyrhizi* isolates

**Fig 2:**
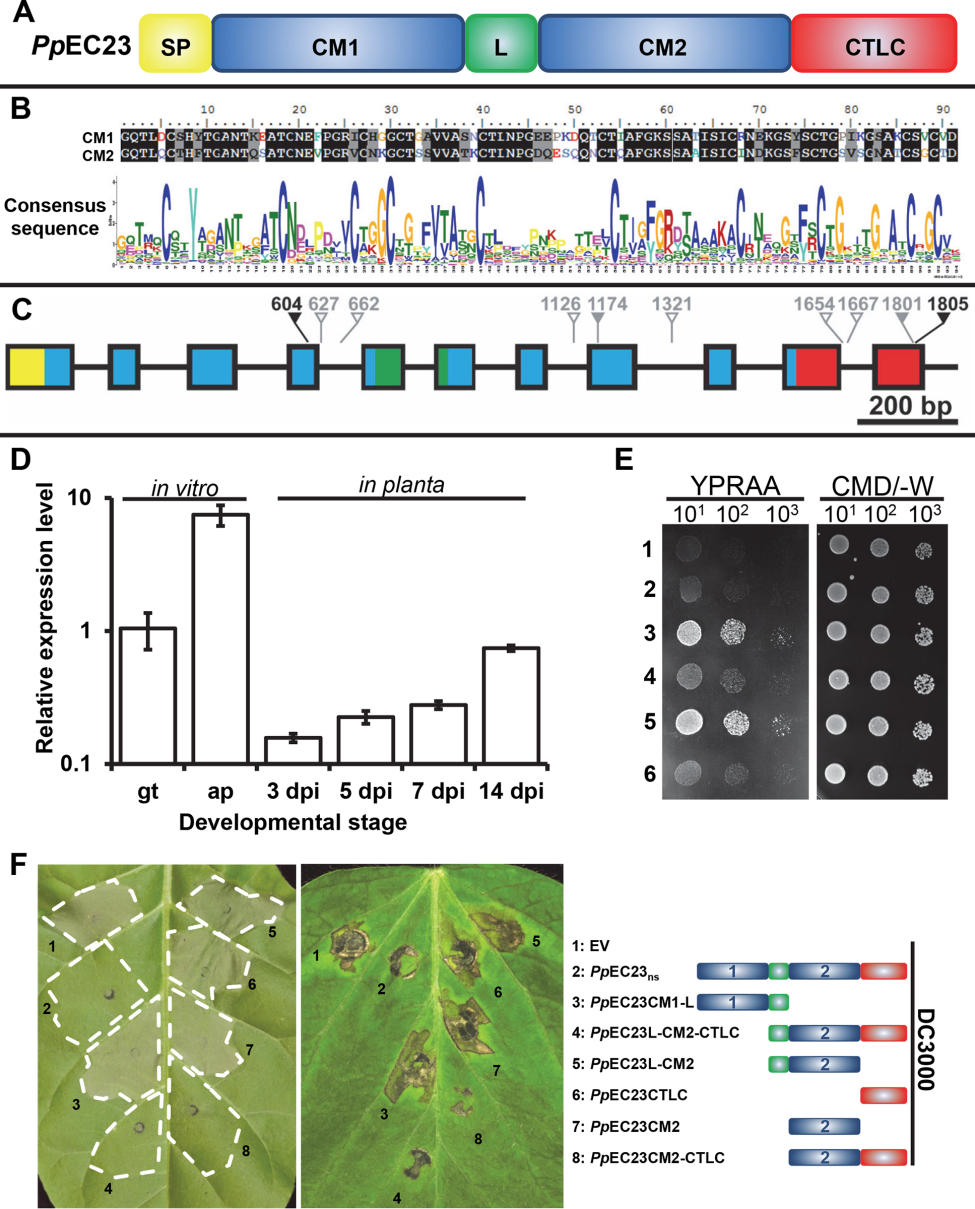
*Pp*EC23 is a modular SSCRP that is present in diverse *P*. *pachyrhizi* isolates. A. Diagram depicting the different motifs of *Pp*EC23. SP, signal peptide; CM1, ten-cysteine motif 1; L, linker; CM2, ten-cysteine motif 2; CTLC, C-terminal low complexity motif. B. Sequence alignment of the two CMs in *Pp*EC23 (top) and the consensus sequence of CM for all members of cluster 112, generated by WebLogo (bottom). C. Genomic structure of the *PpEC23* gene. Colors of the exons shown as blocks correspond with motif colors in panel A. Arrows show the SNPs, together with the nucleotide position, among ten geographically distinct isolates. Hollow gray, solid gray and black arrows show the intron SNPs, synonymous SNPs, and nonsynonymous SNPs, respectively. D. Transcript levels of *Pp*EC23 at different developmental stages. Transcript levels were normalized to the expression of the fungal reference genes, *RPS14* and *PDK*. Three independent biological and four technical replicates were performed. Error bars = standard deviation. E. The *Pp*EC23 signal peptide directs protein secretion in yeast. Fusion constructs of *Pp*EC23 in the secretion signal trap plasmid: 1) pSuc2t7M13ori::*Pp*EC23, 2) pSuc2t7M13ori::*Pp*EC23_ns_, 3) pSuc2t7M13ori::*Pp*EC23Sp-CM1-L, 4) pSuc2t7M13ori::*Pp*EC23L-CM2-CTLC, 5) pSuc2t7M13ori::*Pp*EC23Sp, 6) pSuc2t7M13ori EV control. YPRAA and CMD/-W indicates the selective and non-selective medium, respectively. Numbers below indicate the fold dilution of the yeast strains. F. Symptoms on leaves of *N*. *tabacum* cv. Xanthi (left) and *G*. *max* cv. Williams 82 (middle) infiltrated with *Pst* DC3000 carrying EV, *Pp*EC23_ns_, and various truncated constructs of *Pp*EC23. The name and structural diagram of each construct is provided in the right panel. Leaves were infiltrated with bacteria at OD _600 nm_ = 0.02. Leaves were photographed at 18 hpi for tobacco (left) and at 30 hpi for Williams 82 soybean (middle). Representative images are shown (n ≥ 8).

*Pp*EC23 is a SSCRP containing a signal peptide (SP), two tandem ten-cysteine motifs (CMs) designated CM1 and CM2 separated by a linker region (L), and a C-terminal low complexity (CTLC) domain (Fig 2A). Alignment of the amino acid sequences of CM1 and CM2 showed that they share 65.9% identity (Fig 2B). *Pp*EC23 belongs to a large family of putatively secreted proteins, previously designated as cluster 112 by Link et al. [8], comprised of many SSCRPs from various species of rust fungi. All members contain a single CM, with the exception of *Pp*EC23 which contains two (Fig 2B). The unique structure of *Pp*EC23 and its ability to suppress HR led us to further characterize its properties and functions.

To examine the variability of *PpEC23*, it was PCR-amplified, cloned, and sequenced from the genomic DNA of 11 *P*. *pachyrhizi* isolates collected from ten geographically distinct areas. Four independent clones of each isolate were sequenced and compared to the sequence of isolate LA04-1 [39]. Single nucleotide polymorphisms (SNPs) were found at 10 positions (Fig 2C, Table 1). Eight SNPs were either in introns (hollow gray arrows) or synonymous (solid gray arrows), neither of which altered the coding sequence. Two SNPs, SNP604 and SNP1805, were non-synonymous—generating amino acid changes (black arrows) I125V and A289P, respectively. Eight isolates, including LA04-1, are heterozygous at the SNP1801 and SNP1805 sites. However, isolates BZ01-1, PG01-3, and HW94-1 have more SNPs across 8 of the 10 sites. We calculated an overall SNP density of 5.3 SNPs/kbp, and a non-synonymous SNP density of 1.1 SNPs/kbp in the *Pp*EC23 genomic region, which is dramatically lower than the SNP density of 15.4 SNPs/kbp in the housekeeping genes analyzed previously [40]. The low non-synonymous SNP density of the *Pp*EC23 genomic region shows that its coding sequence is well maintained.

**Table 1:** SNPs of *PpEC23* genes among *P*. *pachyrhizi* isolates.

Isolate	Year	Origin	Source	SNPs*
IN73-1	1973	India	D. N. Thapliyal, Pantnagar	1801T or G; 1805C or G
TW72-1	1972	Taiwan, China	Lung-Chi Wu, Taipei	1801T or G; 1805C or G
TW80-2	1980	Taiwan, China	AVRDC, Taiwan	1801T or G; 1805C or G
BZ01-1	2001	Brazil	J. T. Yorinori, Parana	604A; 627T; 662A; 1126G; 1174A;1321T; 1654G; 1667T; 1801T or G; 1805C
PG01-3	2001	Paraguay	W. M. Morel, Capitan Miranda	604G or A; 627C or T; 662T or A; 1126A or G; 1174G or A; 1321C or T; 1654A or G; 1667C or T; 1801T or G; 1805C or G
HW94-1	1994	Hawaii, US	E. Kilgore, Oahu	604G or A; 627C or T; 662T or A; 1126A or G; 1174G or A; 1321C or T; 1654A or G; 1667C or T; 1805C or G
AU79-1	1979	Australia	Unknown	1801T or G; 1805C or G
SA01-1	2001	South Africa	Z. A. Pretorius, Natal Province	1801T or G; 1805C or G
ZM01-1	2001	Zimbabwe	C. Levy, Harare	1801T or G; 1805C or G
AL04-1	2004	Alabama, US	R. Frederick, Mobile County	1801T or G

*: The genomic sequence of *Pp*EC23 gene from LA04-1 (Louisiana, US) was taken as the reference sequence. Only variations are shown. SNP locations are shown schematically in Fig 2C.

To test if the amino acid differences caused by the two non-synonymous SNPs could affect suppression of *Pst* DC3000-induced HR, SNP604 and SNP1805 were introduced independently into the pEDV6::*Pp*EC23_ns_ sequence, which originated from the LA04-1 isolate. *Pst* DC3000 strains carrying the resulting constructs were infiltrated into soybean (S4 Fig). Both *Pp*EC23 mutants maintained their ability to suppress HR demonstrating that the I125V and A289P amino acid changes did not affect this function.

### The expression pattern of *Pp*EC23

*Pp*EC23 was originally identified by sequencing the *P*. *pachyrhizi* haustorial transcriptome [8], and we were interested in determining its mRNA expression profile throughout the fungal lifecycle. Therefore, we quantified the abundance of *Pp*EC23 mRNA at early infection stages and throughout infection using qRT-PCR. We included *in vitro* produced germ tubes and appressoria, representing the structures that rust fungi form on the leaf surface, and infected leaves at four different time points [3, 5, 7, and 14 days post inoculation (dpi)] using *P. pachyrhizi RPS14* and *PDK* as reference genes [41]. Our analysis shows that transcript levels of *Pp*EC23 are highly elevated during appressoria formation and also continue to rise throughout the infection time course (Fig 2D). This expression profile shows that *Pp*EC23 is not exclusively expressed in haustoria.

### The signal peptide of *Pp*EC23 is sufficient for secretion in a yeast assay

To test the functionality of the predicted signal peptide, we determined if it was able to mediate secretion when fused with invertase in *Saccharomyces cerevisiae*. Expression constructs for fusion proteins containing *Pp*EC23, *Pp*EC23SP, *Pp*EC23_ns_ (the *Pp*EC23 open reading frame minus SP), *Pp*EC23SP-CM1-L and *Pp*EC23L-CM2-CTLC were generated using the yeast signal sequence trap vector pSuc2t7M13ori [42]. The resulting plasmids were transformed into yeast strain YTK12 that lacks a secreted invertase, and transformants were streaked on CMS/loD-W medium, which only supports growth of yeast capable of secreting invertase. As expected, yeast expressing *Pp*EC23SP or *Pp*EC23SP-CM1-L fusions grew well on the selective medium, whereas the transformants expressing the *Pp*EC23_ns_ or *Pp*EC23L-CM2-CTLC fusions lacking the SP showed the same weak growth as the empty vector control (Fig 2E). Transformants expressing full length *Pp*EC23 fused to invertase also grew poorly on selective media. Overall, these results support the functionality of the *Pp*EC23 signal peptide.

### *Pp*EC23 CM2 and CTLC domains are necessary for suppression of HR

To determine the functional domains of *Pp*EC23 required for HR suppression, six truncated constructs consisting of different domain combinations were generated and cloned into pEDV6 (Fig 2F and S5 Fig). *Pst* DC3000 expressing the truncated *Pp*EC23 constructs were inoculated on *N*. *tabacum* and soybean leaves to test if any had the ability to suppress HR (Fig 2F left and middle panels, respectively). Only the *Pp*EC23L-CM2-CTLC and *Pp*EC23CM 2-CTLC constructs retained HR suppression function. These data demonstrate that CM2 and CTLC together are necessary for the ability of *Pp*EC23 to suppress HR induced by *Pst* DC3000.

### *Pp*EC23 suppresses basal defense responses

**Fig 3:**
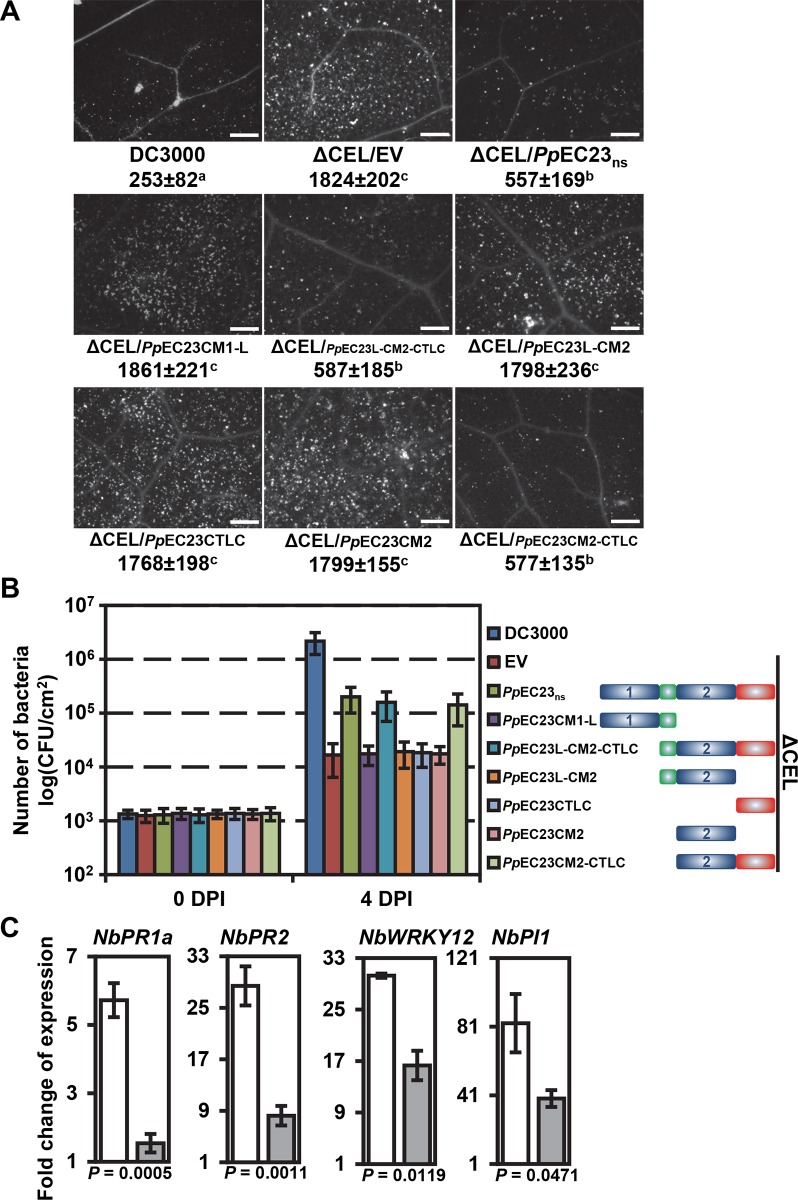
*Pp*EC23 suppresses basal defense responses. A. Callose deposition in leaves of *A*. *thaliana* Col-0 induced by *Pst* DC3000, CUCPB5115 (ΔCEL)/EV, ΔCEL/*Pp*EC23_ns_ or ΔCEL/truncated constructs stained with aniline blue. The average number of callose spots ± standard deviation is listed under each representative image. Pair-wise *t*-tests were performed and a, b, c were designated groups with statistically significant difference. Bar = 50 μm. Representative images are shown (n ≥ 24). B. Bacterial growth *in planta* of *Pst* DC3000, ΔCEL/EV, ΔCEL/*Pp*EC23_ns_ or ΔCEL/truncated constructs. Diagrams of *Pp*EC23_ns_ or truncated constructs of *Pp*EC23 are provided to the right of the graph. Initial inoculum was adjusted uniformly to 10^5^ CFU/mL. Numbers of bacteria were evaluated at 0 dpi and 4 dpi. C. Transcript level fold change of immune marker genes *PR1a*, *PR2*, *WRKY12* and *PI1* in leaves of *N*. *benthamiana* stably transformed with EV (white bars) or FLAG-*Pp*EC23_ns_ (gray bars) at 6 hpi with *P*. *fluorescens* strain EtHAn. *NbAct1* was used as the internal reference gene. *T*-tests were performed for each comparison. The corresponding *P* value is shown in the figure. Four biological and four technical replicates were performed.

Because *Pp*EC23 suppresses HR, we decided to further investigate its function by testing if it also suppresses basal defense responses associated with PTI. *Arabidopsis thaliana* ecotype Columbia-0 (Col-0) normally exhibits extensive callose deposition when inoculated with the *Pst* DC3000 ΔCEL strain CUCPB5115, whereas little callose deposition is observed in leaves inoculated with wild-type *Pst* DC3000 [43,44]. Callose deposition was quantified in *A*. *thaliana* leaves inoculated with *Pst* DC3000 and CUCPB5115 carrying empty pEDV6 or pEDV6 expressing *Pp*EC23_ns_ or its six truncated derivatives (Fig 3A). CUCPB5115 carrying *Pp*EC23_ns_, *Pp*EC23L-CM2-CTLC, or *Pp*EC23CM 2-CTLC induced significantly less callose deposition than CUCPB5115 carrying empty pEDV6, or the other four truncated derivatives of *Pp*EC23. These data show that *Pp*EC23 also suppresses callose deposition and CM2 and CTLC together are necessary for this activity, which is consistent with their function in HR suppression.

Since *Pp*EC23 suppressed basal defense, we expected that it would enhance growth of CUCPB5115 *in planta*. To test this, the same bacterial strains used for the callose deposition assays were inoculated on *A*. *thaliana* (Fig 3B). As expected, wild-type *Pst* DC3000 grew ~100-fold better than CUCPB5115 carrying the empty vector. CUCPB5115 carrying *Pp*EC23_ns_, *Pp*EC23L-CM2-CTLC or *Pp*EC23CM 2-CTLC grew ~10-fold better than CUCPB5115 carrying the empty vector or the other truncated versions of *Pp*EC23. These data show that the ability of *Pp*EC23 to suppress callose deposition is associated with enhanced bacterial growth and that CM2 and CTLC together are necessary for this activity.

The four immune marker genes used in the HR suppression assay can also be used as basal defense markers [45]. Therefore, *N*. *benthamiana* plants transformed with FLAG-*Pp*EC23_ns_ or empty vector were inoculated with EtHAn at OD _600 nm_ of 0.2 to induce PTI. mRNA levels of the four markers genes were examined at 6 hpi using qRT-PCR (Fig 3C). All four marker genes were induced to lower levels in FLAG-*Pp*EC23_ns_ plants, especially *PR1a* and *PR2*, with 3.7 and 3.5 fold lower induction, respectively. The abilities of *Pp*EC23 to suppress callose deposition, promote CUCPB5115 multiplication, and suppress defense gene expression are consistent with a basal defense suppression function.

### *Pp*EC23 interacts with itself

**Fig 4:**
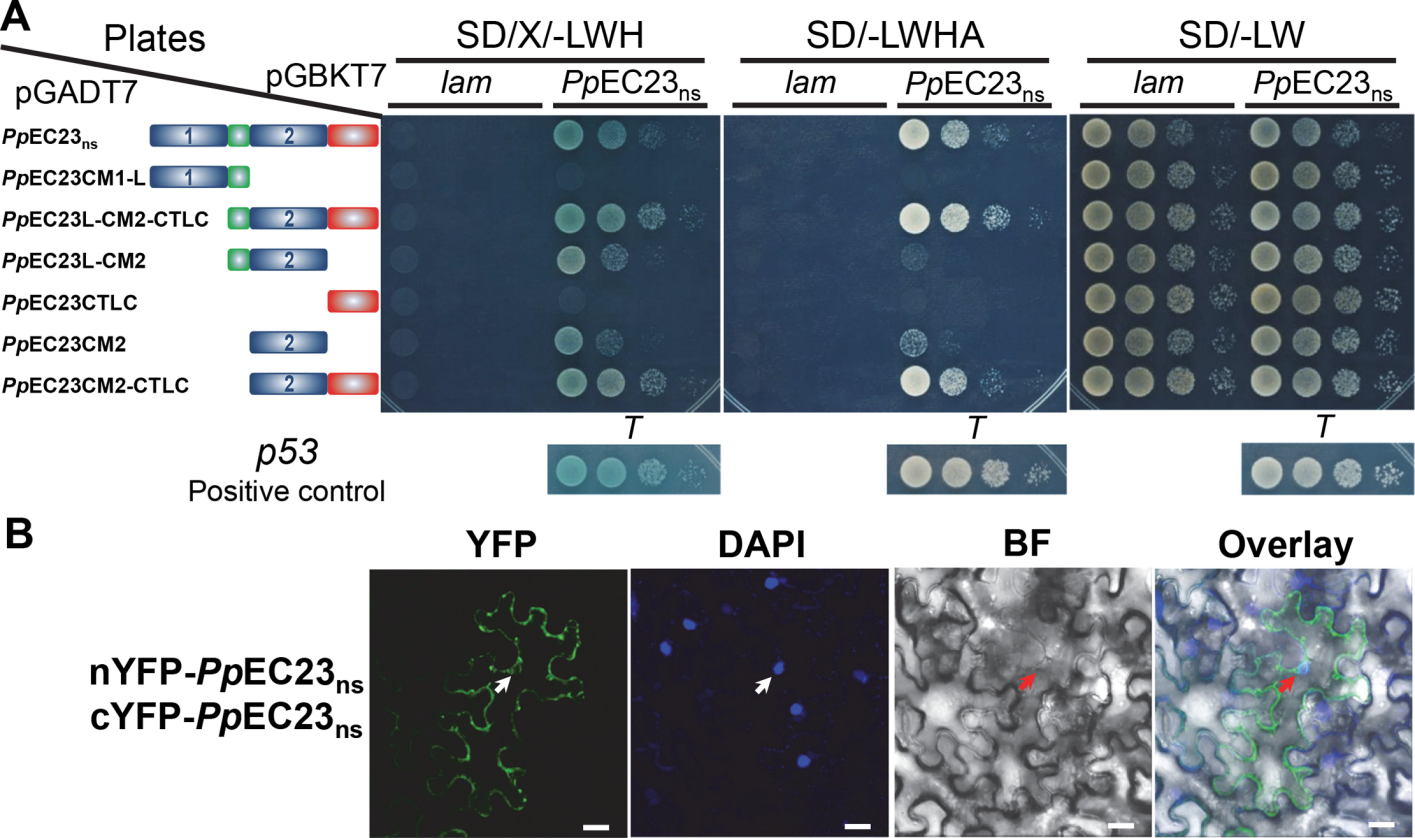
*Pp*EC23 interacts with itself. A. Y2H assay showing that *Pp*EC23 interacts through the C-terminal CM. SD/X/-LWH, SD/-LWHA and SD/-LW represent SD/X-α-gal (-Leu/-Trp/-His), SD (-Leu/-Trp/-His/-Ade) and SD (-Leu/-Trp), respectively. The structural diagrams of *Pp*EC23_ns_ or truncated constructs of *Pp*EC23 are provided next to the relevant strain. The interaction of murine p53 (*p53*) and SV40 large T-antigen (*T*) was used as a positive control for the system, and human lamin C (*lam*) was used as a negative control. B. BiFC assay showing that *Pp*EC23 interacts with itself *in planta*. YFP, yellow fluorescent protein epifluorescence. BF, bright field. DAPI signal was used as a nuclear marker. Arrows indicate nuclei. Representative images are shown (n ≥ 20). Bar = 20 μm.

*Pp*EC23 is a cysteine-rich protein and the cysteine residues in cysteine-rich proteins are reported to form inter- and intramolecular disulfide bridges, which could help to stabilize their ternary structures or to form homodimers or homopolymers [46]. Using a yeast two hybrid assay (Y2H), we found that *Pp*EC23_ns_ does interact with itself (Fig 4A and S6 Fig). To determine the motifs contributing to this self-interaction, we examined the interactions between *Pp*EC23_ns_ and six truncated *Pp*EC23 derivatives (Fig 4A). Ten-fold serially diluted yeast strains were simultaneously cultured on SD (-Leu/-Trp) plates, where all the strains should grow well, and SD/X-α-gal (-Leu/-Trp/-His) and SD (-Leu/-Trp/-His/-Ade) plates, where only interaction-positive strains should grow. *Pp*EC23_ns_ strongly interacted with *Pp*EC23L-CM2-CTLC and *Pp*EC23 CM2-CTLC (Fig 4A). Surprisingly, we also found that *Pp*EC23_ns_ exhibits weaker interactions with *Pp*EC23L-CM2 and *Pp*EC23CM2. Collectively, these data show that CM2 is sufficient for dimerization, while CM2 and CTLC together are necessary for complete interaction.

To test if the *Pp*EC23 self-interaction occurs *in planta*, a bimolecular fluorescence complementation (BiFC) assay was used in *N*. *benthamiana* leaves [47]. For the BiFC assay, the N-terminal half of YFP (nYFP) and the C-terminal half of YFP (cYFP) were fused to the N- or C-terminal end of *Pp*EC23_ns_. Reciprocal combinations of fusion proteins were transiently expressed in *N*. *benthamiana* leaves infiltrated with *Agrobacterium tumefaciens* and were tested for interaction. Representative confocal microscopy images showing *Pp*EC23 dimerization are presented in Fig 4B. We observed strong YFP signals in the cytoplasm, suggesting that *Pp*EC23 protein can also form stable dimers in plant cells with a cytoplasmic subcellular localization (Fig 4B; positive and negative controls for BiFC assays are presented in S7 Fig). Fluorescence could only be observed when the nYFP and cYFP domains were fused to the N terminus of *Pp*EC23_ns_, without disturbing the functional domains in the C terminus. We noticed some YFP signal appeared to be associated with the nucleus. However, based on overlay images, these appear to be surrounding the nucleus. A similar localization pattern was observed when *Pp*EC23 was tagged with full-length GFP, although there also appears to be a significant amount of aggregation of the protein in the cytoplasm (S8 Fig).

### *Pp*EC23 interacts with a soybean transcription factor, *Gm*SPL12l

**Fig 5:**
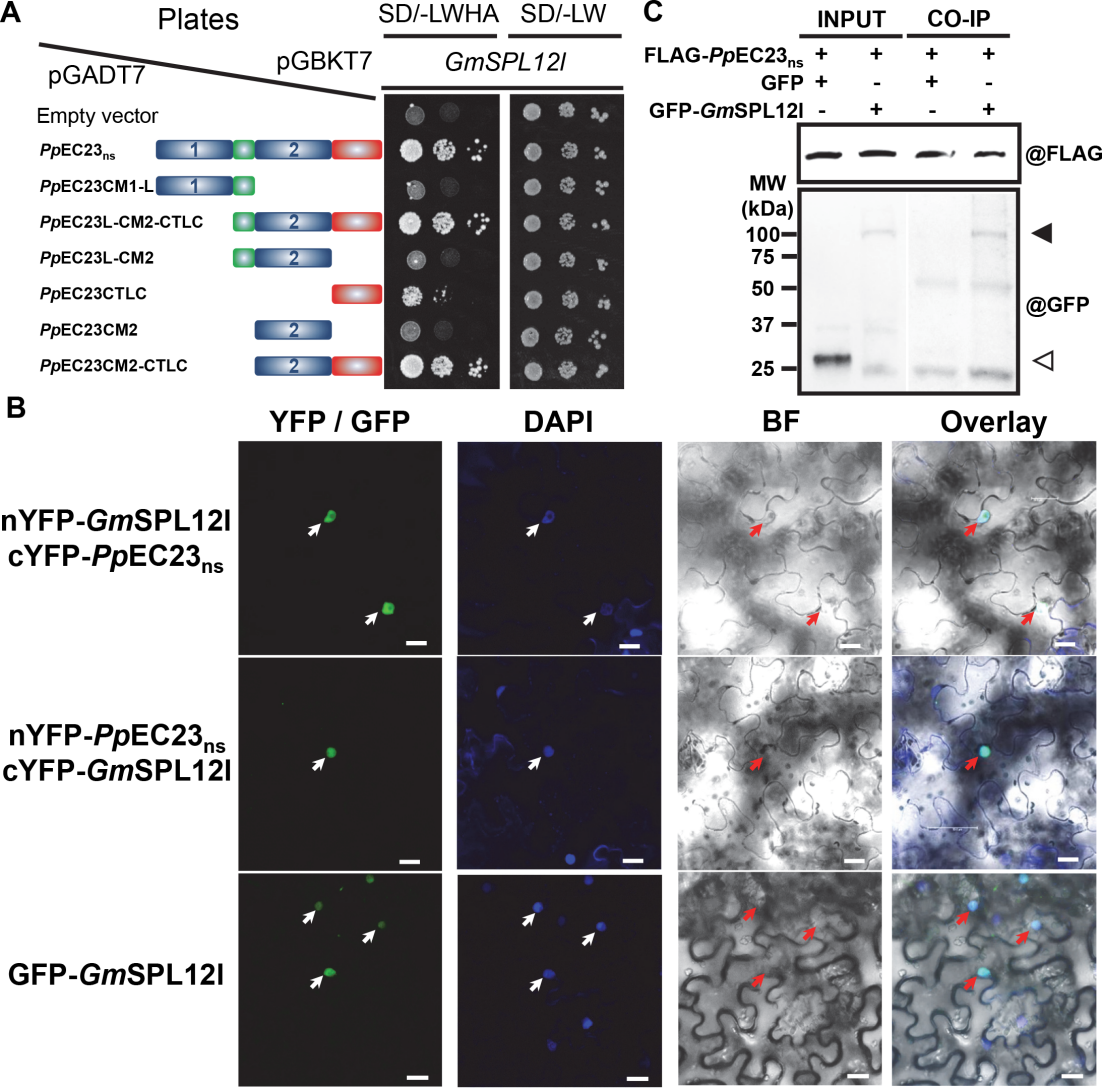
*Pp*EC23 interacts with soybean transcription factor *Gm*SPL12l. A. *Pp*EC23 and *Gm*SPL12l interaction confirmed by Y2H. The empty vector, pGADT7, was included as a negative control. SD/-LWHA and SD/-LW represents SD (-Leu/-Trp/-His/-Ade) and SD (-Leu/-Trp), respectively. The structural diagrams of *Pp*EC23_ns_ or truncated constructs of *Pp*EC23 are shown next to the corresponding strain. B. *Pp*EC23 and *Gm*SPL12l interaction detected in *N*. *benthamiana* nuclei by BiFC assay. YFP/GFP, yellow or green fluorescent protein epifluorescence. BF, bright field. DAPI signal was used as a nuclear marker. Arrows indicate nuclei. Representative images are shown (n ≥ 20). Bar = 20 μm. C. *Pp*EC23 and *Gm*SPL12l interaction confirmed by co-immunoprecipitation assay (CoIP). MW, molecular weight marker. The solid triangle indicates the band corresponding to the GFP-*Gm*SPL12l fusion protein. The white triangle indicates the GFP protein band. @FLAG and @GFP indicate detection using anti-FLAG and anti-GFP antibodies, respectively.

To further investigate the mechanism by which *Pp*EC23_ns_ interferes with plant immune responses, we performed an Y2H screen against a soybean cDNA library. A single interacting protein candidate was identified to be the C-terminal 899 amino acids (aa) of *Gm*SPL12l (Glyma.10g009200), which is a 1,016 aa full-length protein. To confirm the protein-protein interaction observed in yeast, we cloned the full length *GmSPL12l* and performed Y2H against the *Pp*EC23_ns_ and 6 truncated constructs of *Pp*EC23 using the GAL4 Y2H system (Fig 5A). All strains grew well on SD (-Leu/-Trp) plates. On SD (-Leu/-Trp/-His/-Ade) plates, we observed that *Pp*EC23_ns_, together with *Pp*EC23L-CM2-CTLC and *Pp*EC23CM2-CTLC, exhibit strong protein-protein interactions with *Gm*SPL12l. Moreover, we found that *Pp*EC23CTLC weakly interacted with *Gm*SPL12l. These results suggest that CTLC is sufficient for the interaction between *Pp*EC23 and *Gm*SPL12l, while the full interaction requires both CM2 and CTLC.

We then used the BiFC assay to test if the *Pp*EC23 and *Gm*SPL12l interaction could occur *in planta* [47]. nYFP and cYFP were fused to the N or C-terminal ends of the test proteins, *Pp*EC23_ns_ and *Gm*SPL12l, and these fusion proteins were expressed in *N*. *benthamiana* by Agrobacterium infiltration (S9 Fig). Representative confocal microscopy images show that *Pp*EC23 and *Gm*SPL12l can interact with each other (Fig 5B). Co-expression of *Pp*EC23_ns_ and *Gm*SPL12l resulted in bright fluorescence in the nuclei, similar to the localization pattern of GFP*-Gm*SPL12l alone (Fig 5B). Fluorescence was only observed when the nYFP and cYFP domains were fused to the N terminus of *Pp*EC23_ns_ and *Gm*SPL12l, indicating the importance of the C termini of both proteins for the interaction. These data suggest that *Pp*EC23 interacts with *Gm*SPL12l *in planta* and the interaction occurs in nuclei. One Cluster 112 family member from *P*. *pachyrhizi*, *Pp*C112-7, which contains a 10-cysteine-motif [8], and one SPL family member from *A*. *thaliana*, *At*SPL6, which is a positive immune regulator [48], were included to test the specificity of the interaction (S7 Fig). Neither *Pp*C112-7 and *Gm*SPL12l, nor *At*SPL6 and *Pp*EC23_ns_ resulted in detectable fluorescence, indicating that the interaction between *Pp*EC23_ns_ and *Gm*SPL12l is specific.

To further confirm this interaction, we tested if *Pp*EC23 interacts with *Gm*SPL12l in *N*. *benthamiana* extracts using a pull-down assay. GFP or GFP-*Gm*SPL12l was transiently expressed in leaves of the *N*. *benthamiana* lines expressing FLAG-*Pp*EC23_ns_. Total soluble proteins were extracted from leaves at 48 hpi, and FLAG-*Pp*EC23_ns_ was pulled down using anti-FLAG beads. Western blot analysis showed that GFP-*Gm*SPL12l was pulled down together with FLAG-*Pp*EC23_ns_, while GFP, as a negative control, was not pulled down under the same conditions (Fig 5C and S10 Fig). The results demonstrate that FLAG-*Pp*EC23_ns_ physically associates with GFP-*Gm*SPL12l in plant extracts.

Because SPL transcription factors exist widely in many plant species and *Pp*EC23 also suppresses defense in *A*. *thaliana* and *N*. *benthamiana*, we investigated the hypothesis that *Pp*EC23 could interact with SPL homologs in these species by using the BiFC assay. Co-expression of *Nb*SPL1-1 (Niben101Scf01773g04003.1) or *At*SPL1 (AT2G47070) with *Pp*EC23_ns_ resulted in bright fluorescence in the nuclei, similar to the localization pattern of the *Pp*EC23-*Gm*SPL12l interaction (S11 Fig). The interaction specificity between *Pp*EC23 and SPL12 homologs in other non-host plants was confirmed by including the same set of negative control combinations. These data suggested that *Pp*EC23 can also similarly interact with homologs of *Gm*SPL12l in *A*. *thaliana* and *N*. *benthamiana*, suggesting that *Pp*EC23 may use similar mechanisms to suppress the defense responses in non-host and host plants.

### *Gm*SPL12l can suppress the soybean defense responses

**Fig 6:**
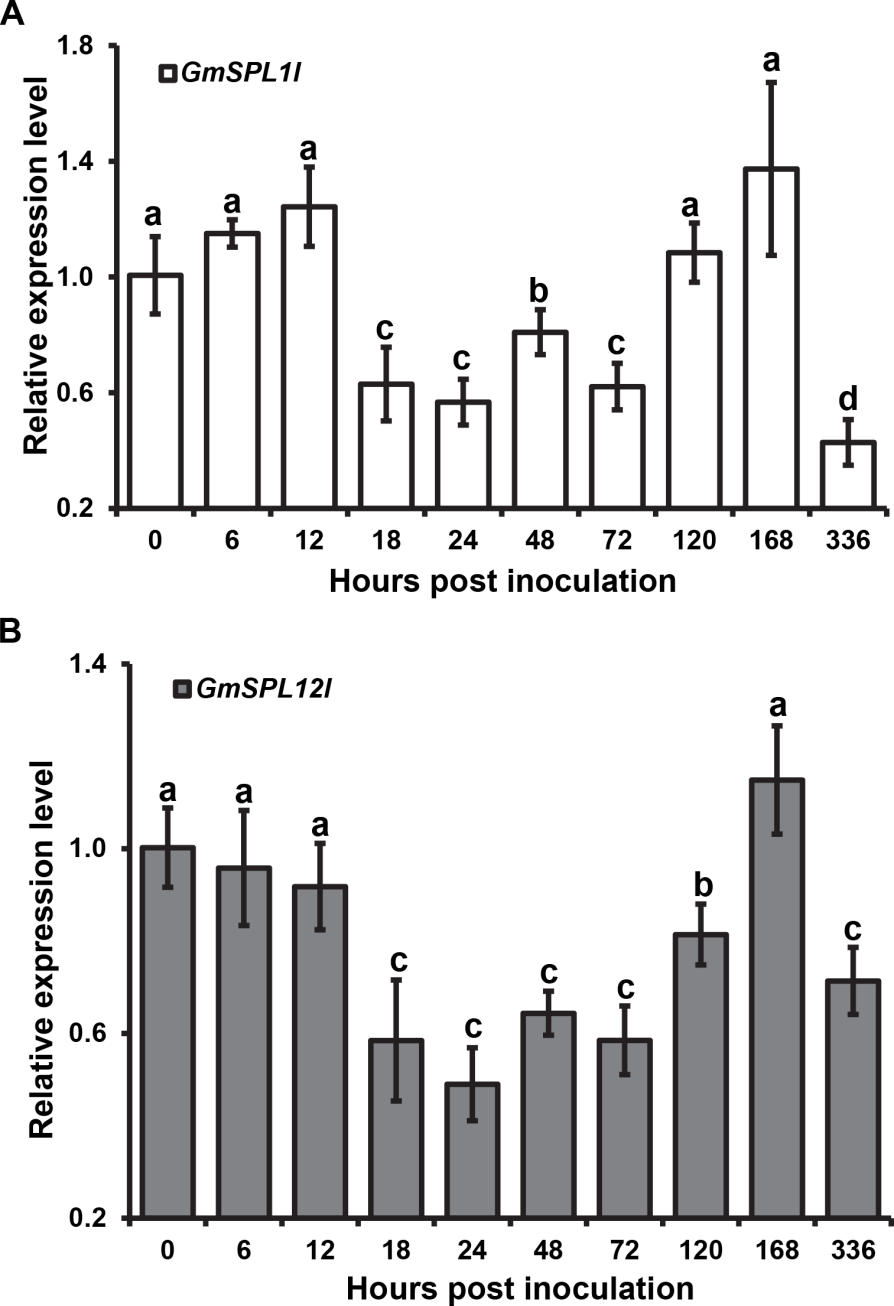
The expression profile of *GmSPL1l* (A) and *GmSPL12l* (B) mRNA during *P*. *pachyrhizi* infection. Expression levels are relative to the expression in soybean without *P*. *pachyrhizi* infection. Three independent biological and four technical replicates were performed. Error bars = standard deviation. Single factor ANOVA and pair-wise two-tailed *t*-test analyses were performed. The letters above the error bars indicate the different groups with statistical significance (*P* < 0.05). The soybean *Ukn2* gene [41] was used as the internal reference gene.

*Gm*SPL12l belongs to the large SQUAMOSA promoter binding-like (SPL) transcription factor family that is involved in many biological processes [49]. In the soybean genome, *GmSPL12l* has a paralogue, *GmSPL1l* (Glyma.02g008600), that differs by only 57 aa. By using qRT-PCR, we found that expression profiles of these two transcription factors are similar during infection (Fig 6). The mRNA transcripts of both genes are down regulated in the time range of 18 to 72 hpi. Afterwards their mRNA levels return to levels comparable to the 0 hpi time point before decreasing again at 336 hpi.

**Fig 7:**
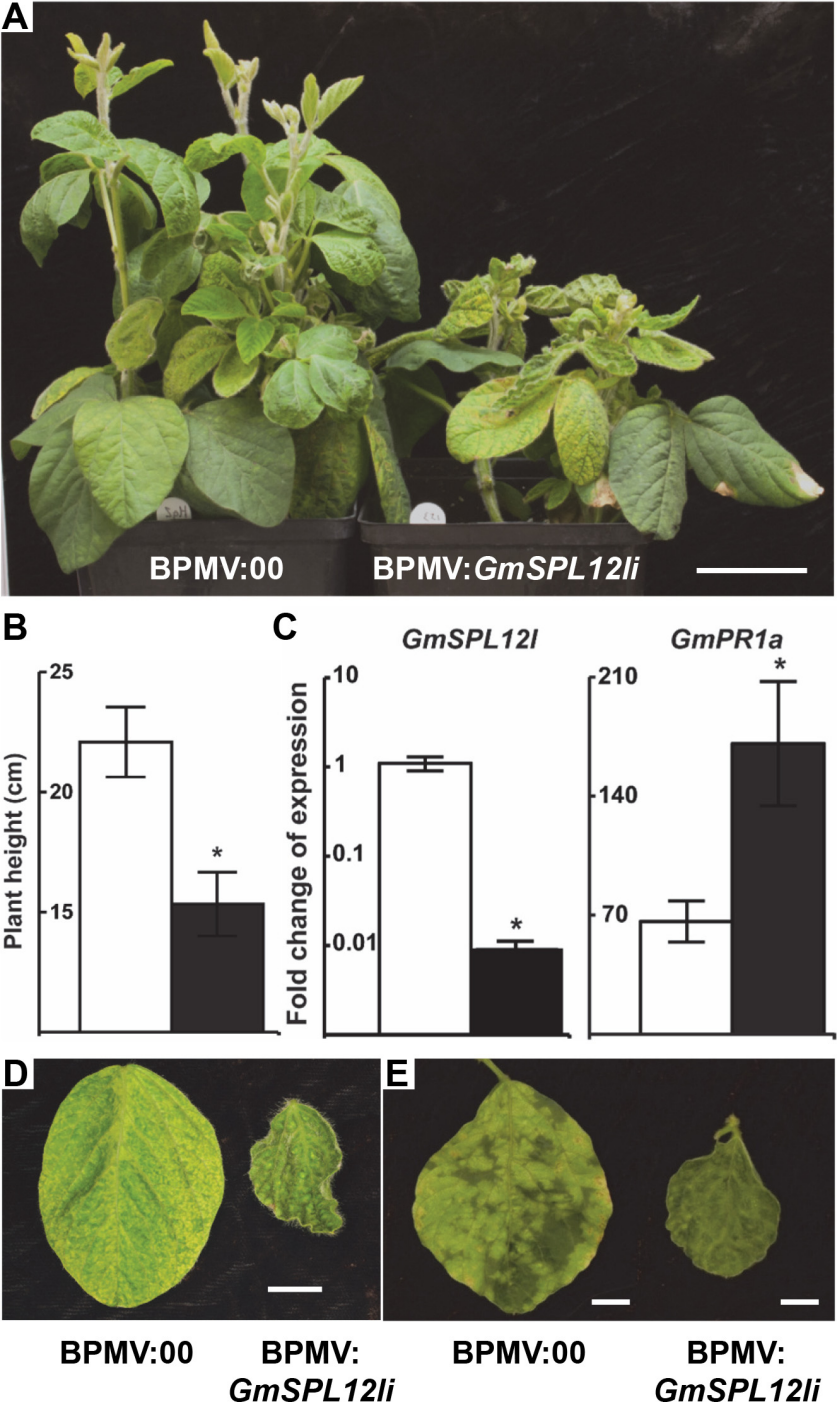
*Gm*SPL12l is a negative regulator of plant immunity. A. Phenotypes of soybean plants 21 dpi with BPMV. A. Soybean plants infected with the BPMV empty vector (BPMV:00) (left) and BPMV:*GmSPL12li* (right). Bar = 5 cm. Representative images are shown (n ≥ 6). B. Heights of plants at 21 dpi with BPMV:00 (white bars) and BPMV:*GmSPL12li* (black bars). A *t*-test was performed for the pair-wise comparison. The * indicates significant difference (*P* < 0.05). Six biological replicates were performed. C. Fold change of *GmSPL12l* (left panel) and *GmPR1a* (right panel) mRNA in BPMV:00 (white bars) and BPMV:*GmSPL12li* (black bars) plants. *GmAct1* was used as the internal reference gene. A *t*-test was performed for each comparison. The * indicates significant difference (*P* < 0.05). Four biological and four technical replicates were performed. D. Representative leaves from BPMV:00 (left) and BPMV:*GmSPL12li* (right) plants (n ≥ 18). Bar = 1 cm. E. Downy mildew symptoms on leaves of BPMV:00 (left) and BPMV:*GmSPL12li* (right) plants inoculated with *P*. *manshurica*. Leaves were photographed at 28 days post BPMV inoculation (7 days post *P*. *manshurica* inoculation). Representative third trifoliate leaves are shown (n ≥ 12). The assay was repeated three times. Bar = 1 cm.

To investigate its role in plant immunity, *GmSPL12l* was silenced using *Bean pod mottle virus* (BPMV)-mediated gene silencing [50]. At 21 dpi, shorter plants with smaller leaves were observed when *GmSPL12l* was silenced compared to the BPMV empty-vector controls (Fig 7A, 7B and 7D). Phenotypes were consistent in three independent replicates. To confirm the silencing of *GmSPL12l*, qRT-PCR was performed to demonstrate that *GmSPL12l* mRNA levels were decreased (Fig 7C). Since *GmSPL1l* is nearly identical in sequence with *GmSPL12l*, we also detected *GmSPL1l* mRNA levels in *GmSPL12l* silencing plants. *GmSPL1l* mRNA levels were also significantly decreased as expected (S12 Fig). We reasoned that the *GmSPL12l* silencing phenotype may have two causes: 1) *GmSPL12l* silencing disturbs plant developmental processes, yielding stunted plants; or 2) *GmSPL12l*-silenced plants have induced defense responses that antagonistically suppress plant growth and development.

To test these hypotheses, *GmSPL12l*-silenced and BPMV empty-vector control plants at 21 dpi were inoculated with the biotrophic pathogen *Peronospora manshurica* (downy mildew). Seven days later, strong chlorotic lesions caused by *P*. *manshurica* infection were observed on leaves of BPMV empty-vector control plants, while no lesions could be observed on the leaves of *GmSPL12l*-silenced plants (Fig 7E). The lack of disease indicated that *GmSPL12l* silencing decreased the susceptibility of soybean to *P*. *manshurica*. We then examined the expression level of *GmPR1a*, a marker gene for soybean defense responses [51], in both BPMV empty-vector control and *GmSPL12l*-silenced plants at 21 dpi using qRT-PCR to determine if defense responses were activated in *GmSPL12l*-silenced plants (Fig 7C). Expression *GmPR1a* was induced 2.6 fold in *GmSPL12l*-silenced plants compared to that in empty-vector control plants, suggesting that *GmSPL12l* silencing significantly activates soybean defense responses. Taken together, these results indicate that *Gm*SPL12l functions as a negative regulator of soybean defense responses.

To test if *N*. *benthamiana* homologs of *Gm*SPL12l might also function as negative regulators of plant immune responses *NbSPL1-1* (Niben101Scf01773g04003.1), *NbSPL1-2* (Niben101Scf09781g00016.1), *NbSPL1-3* (Niben101Scf00687g04018.1) and *NbSPL12* (Niben101Scf02558g00030.1) were silenced using *Tobacco rattle virus* (TRV)-mediated gene silencing [52,53]. Three silencing constructs were designed: *NbSPL1-1/NbSPL1-2i* for silencing both *NbSPL1-1* and *NbSPL1-2*, *NbSPL1-3/SPL12i* for silencing both *NbSPL1-3* and *NbSPL12*, and *NbSPL1si* for silencing all four *NbSPL1*s. At 10 dpi, the TRV-infected plants started to show viral symptoms. There were no obvious differences in morphological or growth phenotypes in the *NbSPL1s* silenced plants, compared to the plants infected with TRV empty vector control. To assess the silencing of *NbSPL1* target genes, qRT-PCR was performed to demonstrate that *NbSPL1* mRNA levels were decreased (S13 Fig). The expression level of *NbSPL1-1/SPL1-2* was decreased in *NbSPL1-1/SPL1-2i* and *NbSPL1si* plants by 64% and 18%, respectively, compared to that of the plants with the TRV empty vector control. The expression level of *NbSPL1-3/SPL12* was decreased in *NbSPL1-3/SPL12i* and *NbSPL1si* plants by 49% and 67%, respectively. These data confirmed the silencing of *NbSPL1*s. To determine the impact of silencing *NbSPL1*s on *N*. *benthamiana* defense responses, the expression levels of the same set of four immune marker genes used in the HR suppression assay were detected by performing qRT-PCR (S13 Fig). The expression levels of all four marker genes were increased in the *NbSPL1* silenced plants, except *NbPI1* in *NbSPL1-1/SPL1-2i* plants. These data indicated that *NbSPL1*s, the homologs of *GmSPL12l* in *N*. *benthamiana*, also act as negative regulators of defense responses, similar to *Gm*SPL12l in soybean.

## Discussion

In this study, we determined that *Pp*EC23 has the ability to suppress HR and basal defense when delivered into plant cells via the T3SS or expressed in transgenic plants. *Pp*EC23 is a modular cysteine-rich protein, in which CM2 and the CTLC domains mediate self-interaction and interaction with a soybean protein. The interacting soybean protein identified is *Gm*SPL12l, a member of the large SPL family of transcription factors. Silencing of *Gm*SPL12l activates mRNA expression of soybean defense genes and enhances resistance to the downy mildew pathogen, *P*. *manshurica*, suggesting that *Pp*EC23 may promote *P*. *pachyrhizi* infection by interacting with a negative regulator of soybean immune responses. The most likely hypothesis postulates that *Pp*EC23 binding alters the stability or activity of *Gm*SPL121, thereby weakening the soybean defenses.

### *Pp*EC23, a SSCRP

Known and predicted effectors from filamentous plant pathogens, including fungi and oomycetes, are often SSCRPs, which are major components of the secretome [54,55]. SSCRPs are normally defined as small proteins (less than 300 aa) with high cysteine content (the total number of cysteine residues representing more than 5% of the mature protein and/or greater than 4 cysteine residues in total), following the predicted secretion signal at the N terminus [55]. SSCRPs from plant pathogens play very important and even decisive roles in the processes of suppression of signal transduction or gene expression in plant cells, protection against antifungal compounds or enzymes. In rust fungi, hundreds of SSCRPs have been predicted, but few of them have been characterized for their roles in virulence [1,8,13,56].

The N-terminal 25 aa of *Pp*EC23 were predicted to encode a putative secretion signal [8], and it was proven functional in a yeast secretion assay (Fig 2E). Excluding the secretion signal, mature *Pp*EC23 is predicted to be 267 aa in total and contains 21 cysteine residues (7.9%), qualifying it as an SSCRP. Furthermore, the cysteine content is as high as 11% within its two tandem repeats of the highly conserved ten-cysteine-motif (Fig 2A). The higher cysteine content significantly increases the number of possibilities for disulfide bridges, which makes the ternary structure of *Pp*EC23 difficult to predict.

Previous work suggested that *Pp*EC23 belongs to a large fungal protein family, Cluster 112, whose members all contain the ten-cysteine motif and most also possess a predicted secretion signal [8]. Sequence alignment showed that the number and positions of the 10 cysteine residues are conserved in all family members, while the intervening amino acid sequences are highly variable, suggesting that the family-specific structure is essentially determined by the number and positions of cysteine residues. The abundance of family members in each rust fungal species implies their importance for these fungi. Notably, all of the family members contain only one copy of the 10-cysteine-motif except for *Pp*EC23, which contains two.

### C-terminal low complexity motif

The C-terminal 59 aa of *Pp*EC23 was predicted as a CTLC motif, which has no reference structures in the structural prediction databases. Thus, the CTLC is expected to have structural flexibility. Surprisingly, we found that the CTLC was sufficient to mediate *Pp*EC23’s interaction with *Gm*SPL12l in the Y2H assay, and this interaction was strengthened by the CM2. Based on this observation, we concluded that the CTLC, together with the CM2, can acquire and maintain the proper structure to interact with *Gm*SPL12l. It was reported that proteins with a terminal low complexity region (LCR) tend to interact with more proteins than those with central LCRs or those without an LCR [57]. Other than in translation and in transport processes, terminal LCR-containing proteins are also enriched in stress-related processes. The N terminal LCR of XopD from *Xanthomonas campestris* pv. *vesicatoria* was demonstrated to enhance bacterial virulence on host plants by forming a coiled-coil-like structure and dimerization [58]. Thus, it is reasonable to expect that the CTLC of *Pp*EC23 interferes with the host plant immune system by interacting with host proteins.

### The genomic sequence of *PpEC23* is well maintained

SNP analyses indicated that nucleotide and aa sequences encoded by *PpEC23* are maintained in diverse *P*. *pachyrhizi* isolates (Fig 2C, Table 1). These isolates were collected from Asia, Africa, South America, and the U.S.A. over a period of approximately 32 years. Most of the isolates, with the exception of BZ01-1, carry two alleles at most of their SNP sites indicating that they are heterozygous. The SNP604, which is only present in BZ01-1, PG01-3, and HW94-1, causes a conservative aa change from isoleucine to valine at position 125, which is in CM1. Based on our analysis of the six *Pp*EC23 truncation mutants, a mutation at this position was not expected to affect ability of *Pp*EC23 to suppress plant immunity or interact with the host target *Gm*SPL12l. SNP1805 is within the CTLC and causes an alanine to proline change, which could potentially have interfered with the function of *Pp*EC23. The introduction of the aa differences in the PpEC23 sequence did not affect its ability to suppress plant immunity.

### *Pp*EC23 suppresses plant immunity

The obligate biotrophic nature of *P*. *pachyrhizi* accompanied by the inability to perform sexual crossings currently limits our ability to directly determine the functions of its genes. To overcome these limitations, we utilized the bacterial secretion vector pEDV6, bacterial strain *Pst* DC3000, and the non-pathogenic *P*. *fluorescens* strain EtHAn to explore the functions of *Pp*EC23 [6,13,29,59,60]. In bacterial inoculation assays, *Pp*EC23 could suppress both HR and basal defense (Figs 1 and 3). *Pp*EC23 was not inherently antimicrobial based on the bacterial growth curve assay (S2 Fig), and its ability to suppress HR was not limited to a single NBS-LRR protein-effector interaction. *Pst* DC3000 causes HR on tobacco and soybean through the recognition of different effectors, HopQ1-1 and AvrD, respectively [30,31]. This observation indicates that *Pp*EC23 functions at a point of convergence downstream of Avr-R recognition that is conserved between legumes and solanaceous plants. The possibility remained that *Pp*EC23 has a direct effect on *Pst* DC3000. However, supplying *Pp*EC23 *in trans* by co-inoculating *Pst* DC3000 with EtHAn strains, or inoculating *Pst* DC3000 on FLAG-*Pp*EC23_ns_ transgenic plants confirmed that HR suppression occurs *in planta* and is not due to an effect that occurred within *Pst* DC3000 (Fig 1B and S1 Fig). Subsequently, we demonstrated that *Pp*EC23 also suppresses basal defense, suggesting that it may interfere with a common node of basal defense and HR signaling. There is increasing evidence that basal defense and HR are not entirely distinct processes [28]. Because *Pp*EC23 affected the expression of the defense marker genes *PR1a*, *PR2*, *WRKY12*, and *PI1* in response to HR and basal defense cues, we expect that it affects regulatory components that are upstream of activation of the expression of these genes. Our data suggests a function for *Pp*EC23 that occurs within cells, which is consistent with its expression in haustoria. However, the finding that it is highly expressed beginning with the formation of appressoria suggests that it could have functions in the apoplast and/or earlier in infection. One of the peculiarities of *P*. *pachyrhizi* is that it does not use stomata to enter into the host, but instead it directly penetrates epidermal cells, which subsequently die [61]. It may be necessary for the fungus to express immune suppressing proteins early to slow cell death or to prevent it from spreading to neighboring cells. It will be interesting to investigate if *PpEC23* may have functions in suppressing host immunity throughout the biotrophic interaction of *P*. *pachyrhizi* and soybean.

### *Gm*SPL12l as a target of *Pp*EC23

SPL proteins constitute a plant-specific family of transcription factors, that are present in single cell algae to higher plants [49]. The SPL family is defined by the conserved SQUAMOSA promoter-binding protein (SBP) domain, consisting of a bipartite nuclear localization signal (NLS) and two, non-interleaved Zn-finger-like structures [62]. SPL proteins are conserved in the SBP domain, which is normally located in the N terminus, but they have diverse C-terminal domains. In the model plant *A*. *thaliana*, SPLs have been well characterized and most are involved in developmental and adaptive programs, including leaf morphogenesis [63], plastochron determination [64], vegetative phase transition [65], flowering [66], anther and gynoecium development [67] and copper homeostasis [68]. *At*SPL6 was recently reported to positively regulate the defense transcriptome following its association with nuclear-localized R proteins [48]. Although the functions of SPL proteins have been analyzed in other plant species, this is the first report of a function for an SPL protein in soybean [69,70]. There are a total of 48 SPL family members predicted in the soybean genome. Sequence analyses of SBP domains suggested that they belong to eight clades along with the 16 *AtS*PLs [71]. *Gm*SPL12l and its paralogue, *Gm*SPL1l, are most closely related to *At*SPL1/12/14/16 according to phylogenetic analyses. However, these *At*SPLs are known to play roles in development but not defense.

We determined that *Gm*SPL12l and *Pp*EC23 interact using three independent assays: Y2H, BiFC, and Co-IP (Fig 5). In these assays, *Gm*SPL12l and *Pp*EC23 interacted only when the epitope tags were fused to the N terminus of both proteins, suggesting the C termini of both proteins are important for protein-protein interaction. Sequence analyses of the C-terminus of *Gm*SPL12l shows that it contains ankyrin-repeats, which normally mediate protein-protein interactions [63]. In the BiFC assay, the interaction of *Gm*SPL12l and *Pp*EC23 was found to localize to the nuclei of plant cells (Fig 5B). Taking into account the cytoplasmic localization of *Pp*EC23 and the nuclear localization of *Gm*SPL12l, the BiFC results show that *Gm*SPL12l can recruit *Pp*EC23 into the nuclei. Furthermore, the observation that the *Pp*EC23 self-interaction was not present in nuclei suggests that does not bind to *Gm*SPL12l as a multimer. The ability of *Pp*EC23 to also interact with *At*SPL1 and *Nb*SPL1-1 in the BiFC assay (S11 Fig) provides a potential mechanism by which it suppresses host immunity in legume, brassica, and solanaceous plants.

*Gm*SPL12l and its paralogue *Gm*SPL1l are highly conserved with only 57 aa differences. The mRNA sequences of these homologous genes, including the 5’- and 3’-UTRs, are also nearly identical. Therefore, our BPMV:*GmPL12li* gene silencing construct silenced both genes, although *GmSPL12l* was silenced ~10 fold more than *GmSPL1l* (Fig 7C and S11 Fig). The similar sequences and expression profiles of the two genes strongly suggest that they may have redundant function, but we have been unable to establish that *Gm*SPL1 can interact with *Pp*EC23 using Y2H. Silencing of *GmSPL12l* and its paralogue resulted in plants that were more resistant to downy mildew and exhibiting a stunted phenotype, suggesting that immunity was activated and plant growth was suppressed (Fig 7). The inverse relationship between immunity and growth is commonly observed in mutants that have elevated or constitutive defense responses, including in soybean [51]. We have performed *P*. *pachyrhizi* infection assays on the *GmSPL12l*-silenced and *PpEC23*-silenced soybeans, however, no change in macroscopic disease phenotypes was observed to date. This negative result is possibly due to the functional redundancy of *P*. *pachyrhizi* effector proteins that suppress immunity. Collectively, our data show that *Gm*SPL12l acts as an immune suppressor possibly to ensure normal plant growth in the absence of infection. However, we postulate that during defense responses, the immune suppression by *Gm*SPL12l could be overcome or dampened to protect the plants from infection.

Transcript levels of *GmSPL12l* and its paralogue decreased after *P*. *pachyrhizi* infection about two fold between 18 and 72 hpi compared to 0 hpi (Fig 6). After 72 hpi, their levels returned to that in non-infected plants suggesting that transcription of *GmSPL12l* is down regulated during early stages of *P*. *pachyrhizi* infection. Because silencing of *GmSPL12l* activated soybean immunity, we expect that it is a negative regulator of plant defenses. Therefore, decreased levels of its transcripts may also lead to activation of soybean defenses during early stages of *P*. *pachyrhizi* infection. The interaction of *Pp*EC23 with *Gm*SPL12l suggests that *Pp*EC23 may manipulate post-translationally or modify *Gm*SPL12l to suppress soybean immunity, which would potentially promote *P*. *pachyrhizi* infection. *PpEC23* mRNA was originally observed in haustoria, but our analyses here showed that it is expressed in pre-infection structures as well as during the infection time course. These data suggest that *Pp*EC23 functions early and throughout the biotrophic interaction between *P*. *pachyrhizi* and soybean, and one of its activities may be to interfere directly with the function or post-translational regulation of *Gm*SPL12l. These will be interesting hypotheses to investigate in the future after we have developed the necessary tools in soybean.

The elevated expression of *PR1a* in *GmSPL12l*-silenced and *NbSPL1-*silenced plants is consistent with our conclusion that *Pp*EC23 targets a protein upstream of defense gene expression at a point of convergence between HR and basal defense, which is conserved between non-host and host plants. However, we do not know if *Gm*SPL12l or *Nb*SPL1 directly or indirectly regulate the expression of defense marker genes such as *PR1a*. In general, the genes that are direct transcriptional targets of the SPLs are not yet known. Studies to investigate this will be aided by stable mutations that knock out *Gm*SPL12l function and development of resources needed for performing chromatin immunoprecipitation coupled with RNA-seq. On the rust side of the interaction, the cluster 112 protein family characterized by the signature ten-cysteine motif is widely present in rust fungal species for which sequence information is available [8]. The presence of this motif suggests that *Pp*EC23 could be a representative of a conserved protein-protein interaction platform that rust fungi use to manipulate host immune responses. It will be interesting to further explore the functions of this family of SSCRPs as virulence factors in rust pathogen-plant interactions.

## Materials and Methods

### Bacterial strains and plasmids

Bacterial strains used in this study are listed in Table 2. *E*. *coli* and *A*. *tumefaciens* were grown in Luria-Bertani (LB) broth at 37°C (*E*. *coli*) or 28°C (*A*. *tumefaciens*) using either liquid or solid media. *Pseudomonas* strains were grown in either LB or King’s B (KB) medium at 28°C. Plasmids were mobilized from *E*. *coli* to *Pseudomonas* strains by standard triparental mating using *E*. *coli* HB101 (pRK2013) as a helper strain.

**Table 2:** Strains and plasmids.

Strain or plasmid	Genotype or relevant phenotype *	Source or reference
*E*. *coli*		
DH5α	F^-^ *end*A1 *gln*V44 *thi*-1 *rec*A1 *rel*A1 *gyr*A96 *deo*R nupG Φ80d*lacZ*ΔM15 Δ(*lacZYA-argF*)U169 *hsd*R17(r_K_^-^ m_K_^+^) λ^–^	Invitrogen
TOP10	F^-^ *mcr*A Δ(*mrr-hsdRMS-mcrBC*) Φ80*lacZ*ΔM15 Δ*lacX74 nup*G *rec*A1 *ara*D139 Δ(*ara-leu*)7697 *gal*E15 *gal*K16 *rps*L(Str^R^) *end*A1 λ^-^	Invitrogen
*P*. *syringae* pv. *tomato*		
DC3000	Wild type, Rif^r^	
CUCPB5115	*∆CEL*::ΩSp/Sm^r^, Rif^r^ Sp^r^	[72]
*P*. *fluorescens*		
EtHAn	*P*. *fluorescens* Pf0-1 carrying a working TTSS from *P*. *syringae* pv. *Syringae*, Cm^r^	[33]
*Agrobacterium tumefaciens*		
GV3101	Carries Vir plasmid encoding T-DNA transfer machinery, Rif^r^, Gm^r^	
*Saccharomyces cerevisiae*		
EGY48	*MATa*, *his3*, *trp1*, *ura3*, *LexA*_*op(x6)*_ *-LEU2*	Clontech
AH109	*MATa*, *trp1-901*, *leu2-3*, *112*, *ura3-52*, *his3-200*, *gal4∆*, *gal80∆*, *LYS2*::*GAL1*_*UAS*_ *-GAL1*_*TATA*_ *-HIS3*, *GAL2*_*UAS*_ *-GAL2*_*TATA*_ *-ADE2*,*URA3*::*MEL1*_*UAS*_ *-MEL1*_*TATA*_ *-lacZ*	Clontech
YTK12	*suc2Δ9 trp1Δ ade2-101 ura3-52*	[42]
Plasmids		
pCR8⁄GW⁄TOPO	Gateway-compatible entry vector, Sp^r^	Invitrogen
pEDV6	Gateway-compatible version of pEDV3, Gm^r^	[29]
pSITEII-3C1	Gateway-compatible binary vector for transiently over-expression of EGFP-fused protein *in planta*, Sm^r^	[73]
pBI121	Binary vector for transformation in plant, Km^r^ in bacteria, Km^r^ in plants	[74]
pSuc2t7M13ori	Yeast signal sequence trap vector, Amp^r^ in bacteria, -Trp in yeast	[42]
pLexA	Bait plasmid for yeast LexA two-hybrid system containing BD domain, Amp^r^ in bacteria, -His in yeast	Clontech
pB42AD	Library plasmid for yeast LexA two-hybrid system containing AD domain, Amp^r^ in bacteria, -Trp in yeast	Clontech
P8op-*lacZ*	LacZ reporter plasmid for yeast LexA two-hybrid system, Amp^r^ in bacteria, -Ura in yeast	Clontech
pGBKT7	Protein expression plasmid for yeast Gal4 two-hybrid system containing BD domain, Km^r^ in bacteria, -Trp in yeast	Clontech
pGADT7	Protein expression plasmid for yeast Gal4 two-hybrid system containing AD domain, Amp^r^ in bacteria, -Leu in yeast	Clontech
pSPYNE-35S	BiFC plasmid containing YFP N-terminal fragment fused to the C-terminus of insertion, Km^r^ in bacteria, Km^r^ in plants	[47]
pSPYCE-35S	BiFC plasmid containing YFP C-terminal fragment fused to the C-terminus of insertion, Km^r^ in bacteria, Bar^r^ in plants	[47]
phygII-SPYNE(R)155	BiFC plasmid containing eYFP N-terminal fragment fused to the N-terminus of insertion, Km^r^ in bacteria, Hyg^r^ in plants	[75]
pkanII-VYCE(R)	BiFC plasmid containing Venus C-terminal fragment fused to the N-terminus of insertion, Km^r^ in bacteria, Km^r^ in plants	[75]
pBPMV-IA-V2	BPMV-based gene silencing vector, Amp^r^	[50]
pYL192	TRV-based gene silencing vector, containing TRV RNA1, Km^r^ in bacteria, Km^r^ in plants	[52]
pYL279	TRV-based gene silencing vector, containing TRV RNA2-Gateway cassette, Km^r^ in bacteria, Km^r^ in plants	[53]
pYL124	TRV-based gene silencing positive control, containing TRV RNA2-*NtPDS*, Km^r^ in bacteria, Km^r^ in plants	[52]

*: Antibiotics concentrations (μg/ml) were used as follows: Rifampicin (Rif) 100, Kanamycin (Km) 75, Gentamycin (Gm) 50, Spectinomycin (Sp) 50, Chloramphenicol (Cm) 30, Ampicillin (Amp) 100, Streptomycin (Sm) 100, Hygromycin B (Hyg) 30 and Basta (Bas) 100.

### Plant and fungal material, inoculation procedure, and production of germ tubes and appressoria

*N*. *benthamiana*, *N*. *tabacum*, and soybean plants were grown in controlled environment chambers at an average temperature of 24°C (range 20°C—26°C), with 45% - 65% relative humidity under long-day conditions (16 h light). *A*. *thaliana* plants were grown in controlled environment chambers at an average temperature of 22°C (range 18°C—24°C), with 45% - 65% relative humidity under short-day conditions (10 h light). For *P*. *pachyrhizi* inoculation, 21 days old soybean plants (cultivar Thorne) were sprayed with a watery suspension containing 0.02% Tween 20, 0.2% milk powder and 0.02% *P*. *pachyrhizi* (isolate Thai1, laboratory collection Universität Hohenheim) urediospores. Plants were kept in the dark over night at 95% humidity and 20°C and then transferred to a greenhouse chamber (day/night 16 h/ 8 h, 22°C). To obtain the germ tube stage, 100 μg urediospores were spread on water in petri dishes and incubated for 12 h at room temperature in the dark. For appressoria, a watery suspension containing 0.02% Tween 20, 0.2% milk powder and 0.02% urediospores was sprayed on a polyethylene membrane and incubated for 16 h in 100% humidity at room temperature in the dark.

### SNP identification

Genomic DNA was extracted from *P*. *pachyrhizi* urediospores using the QIAGEN DNeasy Plant Mini Kit. The *Pp*EC23 genomic sequence was PCR-amplified with Q5 High-Fidelity DNA Polymerase (New England Biolabs) and primers KP680 and KP713 (S1 Table). PCR products were gel purified, modified with 3’-A overhangs using Taq polymerase, and cloned into pCR2.1-TOPO. Clones were sequenced and analyzed using BioEdit (Tom Hall, Ibis Biosciences) to identify SNPs. The SNP density was calculated as the number of SNPs/kb of genomic sequence.

### Bacterial inoculation and growth *in planta*

*Nicotiana* plants used in this study were between 5 and 6 weeks old, *A*. *thaliana* plants were between 4 and 5 weeks old, and soybean plants were 14 days old. All plant assays were performed by infiltrating a bacterial suspension into plant leaves with a needleless syringe. *A*. *tumefaciens* strains were re-suspended in infiltration buffer (100 μM acetosyringone, 10 mM MES, pH 5.6, and 10 mM MgCl_2_) and kept at room temperature for 3 h before infiltration. All other strains were re-suspended in 10 mM MgCl_2_. Areas of bacterial infiltration were marked lightly with a Sharpie permanent marker. Levels of bacterial inoculum used in experiments are noted in the figures. Bacterial levels *in planta* were determined by cutting leaf disks with a cork borer (inner diameter 0.5 cm) and homogenizing them in 500 μl of the inoculation buffer. The resulting suspension containing the bacteria was diluted and plated on KB plates with the appropriate antibiotics.

### Trypan blue staining

Trypan blue staining was performed as described [76] with some minor modifications. Briefly, *N*. *tabacum* and soybean leaves were harvested and incubated in an alcoholic trypan blue lactophenol solution (0.02% trypan blue in the mixture of phenol:glycerol:lactic acid:water:ethanol (1:1:1:1:8 (v/v/v/v))) at 95°C for 5 min, followed by incubation at room temperature for 24 h. Leaf samples were destained using 2.5 g/ml chloral hydrate. Pictures were taken using a Nikon D70 digital camera.

### Callose staining and microscopic analysis

*A*. *thaliana* Col-0 leaves were harvested 12 h after bacterial infiltration, cleared, and stained with aniline blue for callose as described [77]. Leaves were examined with a Zeiss Axioplan II microscope with an A4 fluorescence cube. Numbers of callose depositions were determined with ImageJ software (NIH). Six adjacent fields along the length of the leaf (avoiding the midvein, leaf edge or the syringe-damaged area) were analyzed and averaged. Values in Fig 3 are the average and standard deviation of more than 6 independent leaves for each treatment.

### Yeast secretion assay

Full length or truncated versions of *PpEC23* were cloned into plasmid pSuc2t7M13ori for the yeast signal sequence trap assay [42]. The resulting plasmids were transformed into yeast strain YTK12 using the lithium acetate method [78] without additional carrier DNA. Transformants were plated on CMD/-W medium (0.17% yeast nitrogen base w/o aa, 0.13% dropout mix without tryptophan, 37.8 mM (NH_4_)_2_SO_4_, 2% glucose) [79]. The positive colonies were liquid cultured, adjusted to OD _600 nm_ = 1, serially diluted and plated on YPRAA medium plates (1% yeast extract, 2% bacto peptone, 2% raffinose, 2 ppm antimycin a) [42]. Plates were photographed after 4 days of incubation at 28°C.

### Yeast constructs and two-hybrid screen

The Matchmaker LexA two-hybrid system (Clontech) was used for Y2H screening of a pathogen-infected soybean cDNA library. *PpEC23*_*ns*_ was cloned into the pLexA vector to create a fusion with the DNA BD. Approximately 2.6 × 10^6^ yeast transformants were screened on 2% SD/Gal/Raf/X-β-gal (-Ura/-His/-Trp/-Leu) following the manufacturer’s instructions (Clontech). Direct protein-protein interaction was confirmed by co-transformation of the respective plasmids into the yeast strain AH109 using the Matchmaker GAL4 Two-hybrid System (Clontech), followed by selection of transformants on 2% SD (-Leu/-Trp) at 30°C for 3 days and subsequent transfer to 2% SD/X-α-gal (-Leu/-Trp/-His) and 2% SD (-Leu/-Trp/-His/-Ade) to select for growth of interacting clones. All the relevant constructs were listed in Supplemental Table 1.

### Transient expression of GFP fusion and BiFC constructs in *N*. *benthamiana*

To create GFP-*Gm*SPL12l, the *GmSPL12l* open reading frame was PCR amplified and cloned into the Gateway entry vector pCR8⁄GW⁄TOPO (Invitrogen) and then recombined into the Gateway binary destination vector pSITEII-3C1 (Table 2) using LR clonase II (Invitrogen). For BiFC constructs, *GmSPL12l* and *PpEC23*_*ns*_ were PCR amplified and cloned into the BiFC vectors pSPYNE-35S, pSPYCE-35S, phygII-SPYNE(R)155 and pkanII-VYCE(R) (Table 2). Each protein was independently tagged with either cYFP or nYFP at either the N or C terminus. All the binary vectors were introduced into *A*. *tumefaciens* GV3101 using the freeze-thaw method [80]. GFP or BiFC was detected in *N*. *benthamiana* leaves using a Leica SP5 X MP confocal/multiphoton microscope system at 48 h after infiltration.

### Co-immunoprecipitation assay

For co-immunoprecipitation of *Pp*EC23 and *Gm*SPL12l, *A*. *tumefaciens* containing pSITEII-3C1 (empty vector) or pSITEII-3C1-*Gm*SPL12l were infiltrated into the transgenic *N*. *benthamiana* leaves containing pBI121-FLAG-*Pp*EC23_ns_ (generated at the University of Nebraska Plant Transformation Core Research Facility). Total proteins were extracted as described previously [48]. The pre-cleared protein samples were incubated with anti-FLAG beads (Sigma-Aldrich) at 4°C for 3 h on a rotating shaker and washed 3 times with the buffer. The beads were boiled with 5 × loading buffer (250 mM Tris-Cl, pH 6.8, 10% SDS, 30% glycerol, 5% β-mercaptoethanol and 0.02% bromophenol blue) and samples were separated on an SDS-PAGE gel (4–15%) followed by western blotting using colorimetric alkaline phosphatase (AP) or horseradish peroxidase (HRP) systems.

### RNA isolation and real-time PCR

Approximately 100 mg starting material (leaves, germ tubes, or appressoria) were used for RNA isolation using the RNeasy Plant Mini Kit (Qiagen) according to the manufacturer’s instructions. Two μg of RNA and SuperScript III First Strand kit (Invitrogen) were used for cDNA synthesis. Quantitative real-time PCR (qRT-PCR) was performed using the cDNA and gene-specific primers listed in S1 Table. Each cDNA was amplified by quantitative PCR using iQ SYBR Green Supermix (Bio-Rad) and iCycler real-time PCR system (Bio-Rad). Plant *ACTIN* expression was used to normalize the expression value in each sample in most cases, and the relative expression values were determined against mock samples using the comparative Ct method (2^-∆∆Ct^). For normalization of *P*. *pachyrhizi* genes, *RPS14* and *PDK* [41,81] were used, and in *G*. *max*, *Ukn2* [41,82] was used for normalizing expression of the *GmSPL* genes.

### BPMV-mediated gene silencing

BPMV vector constructs and inoculation of soybean seedlings via biolistic particle bombardment using a Biolistic PDS-1000/He system (Bio-Rad Laboratories, Hercules, CA, USA) have been previously described [50]. A 287 bp fragment from near the 3’ end of the *GmSPL12l* (Glyma.10g009200) open reading frame was amplified by PCR using the primer pair listed in S1 Table, and cloned into pBPMV-V2.

### Downy mildew infection assay

Downy mildew infection was performed as described previously [83]. The field isolate of *P*. *manshurica* was maintained on soybean plants in the greenhouse. BPMV-infected soybean plants were spray-inoculated with the sporangia suspension (10^4^ sporangia/ ml) of *P*. *manshurica*. Plants were kept overnight in the dark with high humidity and then moved to the growth chamber for 7 days. Symptoms of the top leaves were observed and recorded with a digital camera.

## Supporting Information

S1 Fig Confirmation that *Pp*EC23 suppresses *Pst* DC3000 induced HR in *N*. *benthamiana*. (TIF)

S2 Fig *Pp*EC23 has no direct antimicrobial effect on *Pst* DC3000. (TIF)

S3 Fig Expression of the FLAG-*Pp*EC23 fusion protein in transgenic *N*. *benthamiana* lines. (TIF)

S4 Fig The amino acid changes due to the non-synonymous SNPs have no impact on the *Pp*EC23 function of suppressing HR. (TIF)

S5 Fig Expression of truncated *Pp*EC23 constructs confirmed by PCR and western blot. (TIF)

S6 Fig Confirmation of protein expression in yeast strains. (TIF)

S7 Fig Positive and negative controls for BiFC assays. (TIF)

S8 Fig Subcellular localization of GFP-*Pp*EC23_ns_
(TIF)

S9 Fig Confirmation of protein expression from the BiFC constructs. (TIF)

S10 Fig Co-IP of *Pp*EC23 and *Gm*SPL12l in three independent, transgenic *N*. *benthamiana* lines expressing FLAG-*Pp*EC23_ns_. (TIF)

S11 Fig *Gm*SPL12l homologs from *A*. *thaliana* and *N*. *benthamiana* can also interact with *Pp*EC23. (TIF)

S12 Fig Fold change of *GmSPL1l* mRNA in BPMV:00 (white bars) and BPMV:*GmSPL12li* (black bars) plants. (TIF)

S13 Fig The effects of silencing *NbSPL1* homologs on the expression of immune marker genes *PR1a*, *PR2*, *WRKY12*, and *PI1* in *N*. *benthamiana* plants. (TIF)

S1 Table Primers used in this study. (DOCX)

## References

[1] Duplessis S, Cuomo CA, Lin Y-C, Aerts A, Tisserant E, et al (2011) Obligate biotrophy features unraveled by the genomic analysis of rust fungi. Proc Natl Acad Sci U S A 108: 9166–9171. 10.1073/pnas.1019315108 21536894 PMC3107277

[2] Petre B, Joly DL, Duplessis S (2014) Effector proteins of rust fungi. Front Plant Sci 5 10.3389/fpls.2014.00416 PMC4139122 25191335

[3] Nemri A, Saunders DGO, Anderson C, Upadhyaya NM, Win J, et al (2014) The genome sequence and effector complement of the flax rust pathogen *Melampsora lini* . Front Plant Sci 5: 98 10.3389/fpls.2014.00098 24715894 PMC3970004

[4] Voegele RT, Mendgen KW (2011) Nutrient uptake in rust fungi: how sweet is parasitic life? Euphytica 179: 41–55. 10.1007/s10681-011-0358-5

[5] Rafiqi M, Ellis JG, Ludowici VA, Hardham AR, Dodds PN (2012) Challenges and progress towards understanding the role of effectors in plant-fungal interactions. Curr Opin Plant Biol 15: 477–482. 10.1016/j.pbi.2012.05.003 22658704

[6] Kemen E, Gardiner A, Schultz-Larsen T, Kemen AC, Balmuth AL, et al (2011) Gene gain and loss during evolution of obligate parasitism in the white rust pathogen of *Arabidopsis thaliana* . PLoS Biol 9: e1001094 10.1371/journal.pbio.1001094 21750662 PMC3130010

[7] Cantu D, Segovia V, MacLean D, Bayles R, Chen X, et al (2013) Genome analyses of the wheat yellow (stripe) rust pathogen *Puccinia striiformis* f. sp. *tritici* reveal polymorphic and haustorial expressed secreted proteins as candidate effectors. BMC Genomics 14: 270 10.1186/1471-2164-14-270 23607900 PMC3640902

[8] Link TI, Lang P, Scheffler BE, Duke M V, Graham MA, et al (2014) The haustorial transcriptomes of *Uromyces appendiculatus* and *Phakopsora pachyrhizi* and their candidate effector families. Mol Plant Pathol 15: 379–393. 10.1111/mpp.12099 24341524 PMC6638672

[9] Pretsch K, Kemen A, Kemen E, Geiger M, Mendgen K, et al (2013) The rust transferred proteins-a new family of effector proteins exhibiting protease inhibitor function. Mol Plant Pathol 14: 96–107. 10.1111/j.1364-3703.2012.00832.x 22998218 PMC6638633

[10] Ellis JG, Dodds PN, Lawrence GJ (2007) Flax rust resistance gene specificity is based on direct resistance-avirulence protein interactions. Annu Rev Phytopathol 45: 289–306. 10.1146/annurev.phyto.45.062806.094331 17430087

[11] Kemen E, Kemen AC, Rafiqi M, Hempel U, Mendgen K, et al (2005) Identification of a protein from rust fungi transferred from haustoria into infected plant cells. Mol Plant Microbe Interact 18: 1130–1139. 10.1094/MPMI-18-1130 16353548

[12] Upadhyaya NM, Mago R, Staskawicz BJ, Ayliffe MA, Ellis JG, et al (2014) A bacterial type III secretion assay for delivery of fungal effector proteins into wheat. Mol Plant Microbe Interact 27: 255–264. 10.1094/MPMI-07-13-0187-FI 24156769

[13] Liu C, Pedersen C, Schultz-Larsen T, Aguilar GB, Madriz-Ordeñana K, et al (2016) The stripe rust fungal effector PEC6 suppresses pattern-triggered immunity in a host species-independent manner and interacts with adenosine kinases. New Phytol. 10.1111/nph.14034 27252028

[14] Catanzariti A-M, Dodds PN, Lawrence GJ, Ayliffe MA, Ellis JG (2006) Haustorially expressed secreted proteins from flax rust are highly enriched for avirulence elicitors. Plant Cell 18: 243–256. 10.1105/tpc.105.035980 16326930 PMC1323496

[15] Kale SD, Gu B, Capelluto DGS, Dou D, Feldman E, et al (2010) External lipid PI3P mediates entry of eukaryotic pathogen effectors into plant and animal host cells. Cell 142: 981–983. 10.1016/j.cell.2010.08.035 20655469

[16] Rafiqi M, Gan PHP, Ravensdale M, Lawrence GJ, Ellis JG, et al (2010) Internalization of flax rust avirulence proteins into flax and tobacco cells can occur in the absence of the pathogen. Plant Cell 22: 2017–2032. 10.1105/tpc.109.072983 20525849 PMC2910983

[17] Goellner K, Loehrer M, Langenbach C, Conrath U, Koch E, et al (2010) *Phakopsora pachyrhizi*, the causal agent of Asian soybean rust. Mol Plant Pathol 11: 169–177. 10.1111/j.1364-3703.2009.00589.x 20447267 PMC6640291

[18] Harris DK, Kendrick MD, King ZR, Pedley KF, Walker DR, et al (2015) Identification of unique genetic sources of soybean rust resistance from the USDA soybean germplasm collection. Crop Sci.

[19] Hartman GL, Miles MR, Frederick RD (2005) Breeding for resistance to soybean rust. Plant Dis 89: 664–666. 10.1094/PD-89-0664 30795395

[20] Duplessis S, Joly DL, Dodds PN (2012) Rust effectors In Effectors in plant-microbe interactions. Martin F, Kamoun S, editors West Sussex, UK: John Wiley & Sons 155–193 p. 10.1002/9781119949138.ch7

[21] Chisholm ST, Coaker G, Day B, Staskawicz BJ (2006) Host-microbe interactions: shaping the evolution of the plant immune response. Cell 124: 803–814. 10.1016/j.cell.2006.02.008 16497589

[22] Jones JDG, Dangl JL (2006) The plant immune system. Nature 444: 323–329. 10.1038/nature05286 17108957

[23] Zipfel C, Robatzek S, Navarro L, Oakeley EJ, Jones JDG, et al (2004) Bacterial disease resistance in Arabidopsis through flagellin perception. Nature 428: 764–767. 10.1038/nature02485 15085136

[24] Zipfel C, Kunze G, Chinchilla D, Caniard A, Jones JDG, et al (2006) Perception of the bacterial PAMP EF-Tu by the receptor EFR restricts Agrobacterium-mediated transformation. Cell 125: 749–760. 10.1016/j.cell.2006.03.037 16713565

[25] Ade J, DeYoung BJ, Golstein C, Innes RW (2007) Indirect activation of a plant nucleotide binding site-leucine-rich repeat protein by a bacterial protease. Proc Natl Acad Sci U S A 104: 2531–2536. 10.1073/pnas.0608779104 17277084 PMC1790868

[26] Schwessinger B, Zipfel C (2008) News from the frontline: recent insights into PAMP-triggered immunity in plants. Curr Opin Plant Biol 11: 389–395. 10.1016/j.pbi.2008.06.001 18602859

[27] Dangl JL, Horvath DM, Staskawicz BJ (2013) Pivoting the plant immune system from dissection to deployment. Science 341: 746–751. 10.1126/science.1236011 23950531 PMC3869199

[28] Thomma BPHJ, Nürnberger T, Joosten MHAJ (2011) Of PAMPs and effectors: the blurred PTI-ETI dichotomy. Plant Cell 23: 4–15. 10.1105/tpc.110.082602 21278123 PMC3051239

[29] Sohn KH, Lei R, Nemri A, Jones JDG (2007) The downy mildew effector proteins ATR1 and ATR13 promote disease susceptibility in *Arabidopsis thaliana* . Plant Cell 19: 4077–4090. 10.1105/tpc.107.054262 18165328 PMC2217653

[30] Kobayashi DY, Tamaki SJ, Keen NT (1989) Cloned avirulence genes from the tomato pathogen *Pseudomonas syringae* pv. *tomato* confer cultivar specificity on soybean. Proc Natl Acad Sci U S A 86: 157–161. 10.1073/pnas.86.1.157 16578838 PMC286423

[31] Wei C-F, Kvitko BH, Shimizu R, Crabill E, Alfano JR, et al (2007) A *Pseudomonas syringae* pv. *tomato* DC3000 mutant lacking the type III effector HopQ1-1 is able to cause disease in the model plant *Nicotiana benthamiana* . Plant J 51: 32–46. 10.1111/j.1365-313X.2007.03126.x 17559511

[32] Katagiri F, Thilmony R, He SY (2002) The *Arabidopsis thaliana*-*Pseudomonas syringae* interaction. Arabidopsis Book 1: e0039 10.1199/tab.0039 22303207 PMC3243347

[33] Thomas WJ, Thireault CA, Kimbrel JA, Chang JH (2009) Recombineering and stable integration of the *Pseudomonas syringae* pv. *syringae* 61 hrp/hrc cluster into the genome of the soil bacterium *Pseudomonas fluorescens* Pf0-1. Plant J 60: 919–928. 10.1111/j.1365-313X.2009.03998.x 19682294

[34] Sels J, Mathys J, De Coninck BMA, Cammue BPA, De Bolle MFC (2008) Plant pathogenesis-related (PR) proteins: a focus on PR peptides. Plant Physiol Biochem 46: 941–950. 10.1016/j.plaphy.2008.06.011 18674922

[35] Wang H, Avci U, Nakashima J, Hahn MG, Chen F, et al (2010) Mutation of WRKY transcription factors initiates pith secondary wall formation and increases stem biomass in dicotyledonous plants. Proc Natl Acad Sci U S A 107: 22338–22343. 10.1073/pnas.1016436107 21135241 PMC3009815

[36] Kim HS, Park YH, Nam H, Lee YM, Song K, et al (2014) Overexpression of the *Brassica rapa* transcription factor WRKY12 results in reduced soft rot symptoms caused by *Pectobacterium carotovorum* in Arabidopsis and Chinese cabbage. Plant Biol 16: 973–981. 10.1111/plb.12149 24552622

[37] Haq SK, Atif SM, Khan RH (2004) Protein proteinase inhibitor genes in combat against insects, pests, and pathogens: natural and engineered phytoprotection. Arch Biochem Biophys 431: 145–159. 10.1016/j.abb.2004.07.022 15464737

[38] Valueva TA, Mosolov V V. (2004) Role of inhibitors of proteolytic enzymes in plant defense against phytopathogenic microorganisms. Biochem 69: 1305–1309. 10.1007/s10541-005-0015-5 15627384

[39] Baysal-Gurel F, Ivey MLL, Dorrance A, Luster D, Frederick R, et al (2008) An immunofluorescence assay to detect urediniospores of *Phakopsora pachyrhizi* . Plant Dis 92: 1387–1393. 10.1094/PDIS-92-10-1387 30769566

[40] Zhang XC, Freire MCM, Le MH, De Oliveira LO, Pitkin JW, et al (2012) Genetic diversity and origins of *Phakopsora pachyrhizi* isolates in the United States. Asian J Plant Pathol 6: 52–65. 10.3923/ajppaj.2012.52.65

[41] Hirschburger D, Müller M, Voegele RT, Link T (2015) Reference genes in the pathosystem *Phakopsora pachyrhizi*/soybean suitable for normalization in transcript profiling. Int J Mol Sci 16: 23057–23075. 10.3390/ijms160923057 26404265 PMC4613351

[42] Jacobs KA, Collins-Racie LA, Colbert M, Duckett M, Golden-Fleet M, et al (1997) A genetic selection for isolating cDNAs encoding secreted proteins. Gene 198: 289–296. 10.1016/S0378-1119(97)00330-2 9370294

[43] Alfano JR, Charkowski AO, Deng W-L, Badel JL, Petnicki-Ocwieja T, et al (2000) The *Pseudomonas syringae* Hrp pathogenicity island has a tripartite mosaic structure composed of a cluster of type III secretion genes bounded by exchangeable effector and conserved effector loci that contribute to parasitic fitness and pathogenicity in plants. Proc Natl Acad Sci 97: 4856–4861. 10.1073/pnas.97.9.4856 10781092 PMC18322

[44] DebRoy S, Thilmony R, Kwack Y-B, Nomura K, He SY (2004) A family of conserved bacterial effectors inhibits salicylic acid-mediated basal immunity and promotes disease necrosis in plants. Proc Natl Acad Sci U S A 101: 9927–9932. 10.1073/pnas.0401601101 15210989 PMC470775

[45] Liu Y, Wang L, Cai G, Jiang S, Sun L, et al (2013) Response of tobacco to the *Pseudomonas syringae* pv. *tomato* DC3000 is mainly dependent on salicylic acid signaling pathway. FEMS Microbiol Lett 344: 77–85. 10.1111/1574-6968.12157 23581479

[46] Rep M (2005) Small proteins of plant-pathogenic fungi secreted during host colonization. FEMS Microbiol Lett 253: 19–27. 10.1016/j.femsle.2005.09.014 16216445

[47] Walter M, Chaban C, Schütze K, Batistic O, Weckermann K, et al (2004) Visualization of protein interactions in living plant cells using bimolecular fluorescence complementation. Plant J 40: 428–438. 10.1111/j.1365-313X.2004.02219.x 15469500

[48] Padmanabhan MS, Ma S, Burch-Smith TM, Czymmek K, Huijser P, et al (2013) Novel positive regulatory role for the SPL6 transcription factor in the N TIR-NB-LRR receptor-mediated plant innate immunity. PLoS Pathog 9: e1003235 10.1371/journal.ppat.1003235 23516366 PMC3597514

[49] Chen X, Zhang Z, Liu D, Zhang K, Li A, et al (2010) SQUAMOSA promoter-binding protein-like transcription factors: star players for plant growth and development. J Integr Plant Biol 52: 946–951. 10.1111/j.1744-7909.2010.00987.x 20977652

[50] Zhang C, Bradshaw JD, Whitham SA, Hill JH (2010) The development of an efficient multipurpose *Bean pod mottle virus* viral vector set for foreign gene expression and RNA silencing. Plant Physiol 153: 52–65. 10.1104/pp.109.151639 20200069 PMC2862437

[51] Liu J-Z, Braun E, Qiu W-L, Shi Y-F, Marcelino-Guimarães FC, et al (2014) Positive and negative roles for soybean MPK6 in regulating defense responses. Mol Plant Microbe Interact 27: 824–834. 10.1094/MPMI-11-13-0350-R 24762222

[52] Liu Y, Schiff M, Marathe R, Dinesh-Kumar SP (2002) Tobacco *Rar1*, *EDS1* and *NPR1/NIM1* like genes are required for *N*-mediated resistance to *Tobacco mosaic virus* . Plant J 30: 415–429. 10.1046/j.1365-313X.2002.01297.x 12028572

[53] Liu Y, Schiff M, Dinesh-Kumar SP (2002) Virus-induced gene silencing in tomato. Plant J 31: 777–786. 10.1046/j.1365-313X.2002.01394.x 12220268

[54] Stergiopoulos I, de Wit PJGM (2009) Fungal effector proteins. Annu Rev Phytopathol 47: 233–263. 10.1146/annurev.phyto.112408.132637 19400631

[55] Krijger J-J, Thon MR, Deising HB, Wirsel SGR (2014) Compositions of fungal secretomes indicate a greater impact of phylogenetic history than lifestyle adaptation. BMC Genomics 15: 722 10.1186/1471-2164-15-722 25159997 PMC4161775

[56] Saunders DGO, Win J, Cano LM, Szabo LJ, Kamoun S, et al (2012) Using hierarchical clustering of secreted protein families to classify and rank candidate effectors of rust fungi. PLoS One 7: e29847 10.1371/journal.pone.0029847 22238666 PMC3253089

[57] Coletta A, Pinney JW, Solís DYW, Marsh J, Pettifer SR, et al (2010) Low-complexity regions within protein sequences have position-dependent roles. BMC Syst Biol 4: 43 10.1186/1752-0509-4-43 20385029 PMC2873317

[58] Canonne J, Marino D, Noël LD, Arechaga I, Pichereaux C, et al (2010) Detection and functional characterization of a 215 amino acid N-terminal extension in the Xanthomonas type III effector XopD. PLoS One 5: e15773 10.1371/journal.pone.0015773 21203472 PMC3008746

[59] Cabral A, Stassen JHM, Seidl MF, Bautor J, Parker JE, et al (2011) Identification of *Hyaloperonospora arabidopsidis* transcript sequences expressed during infection reveals isolate-specific effectors. PLoS One 6: e19328 10.1371/journal.pone.0019328 21573066 PMC3090399

[60] Fabro G, Steinbrenner J, Coates M, Ishaque N, Baxter L, et al (2011) Multiple candidate effectors from the oomycete pathogen *Hyaloperonospora arabidopsidis* suppress host plant immunity. PLoS Pathog 7: e1002348 10.1371/journal.ppat.1002348 22072967 PMC3207932

[61] Koch E, Ebrahim Nesbat F, Hoppe HH (1983) Light and electron microscopic studies on the development of soybean rust (*Phakopsora pachyrhizi* Syd.) in susceptible soybean leaves [*Glycine max*]. Phytopathol Zeitschrift 106: 302–320.

[62] Yamasaki K, Kigawa T, Inoue M, Tateno M, Yamasaki T, et al (2004) A novel zinc-binding motif revealed by solution structures of DNA-binding domains of Arabidopsis SBP-family transcription factors. J Mol Biol 337: 49–63. 10.1016/j.jmb.2004.01.015 15001351

[63] Stone JM, Liang X, Nekl ER, Stiers JJ (2005) Arabidopsis *At*SPL14, a plant-specific SBP-domain transcription factor, participates in plant development and sensitivity to fumonisin B1. Plant J 41: 744–754. 10.1111/j.1365-313X.2005.02334.x 15703061

[64] Wang J-W, Schwab R, Czech B, Mica E, Weigel D (2008) Dual effects of miR156-targeted *SPL* genes and *CYP78A5/KLUH* on plastochron length and organ size in *Arabidopsis thaliana* . Plant Cell 20: 1231–1243. 10.1105/tpc.108.058180 18492871 PMC2438454

[65] Shikata M, Koyama T, Mitsuda N, Ohme-Takagi M (2009) Arabidopsis SBP-box genes *SPL10*, *SPL11* and *SPL2* control morphological change in association with shoot maturation in the reproductive phase. Plant Cell Physiol 50: 2133–2145. 10.1093/pcp/pcp148 19880401

[66] Wang J-W, Czech B, Weigel D (2009) miR156-regulated SPL transcription factors define an endogenous flowering pathway in *Arabidopsis thaliana* . Cell 138: 738–749. 10.1016/j.cell.2009.06.014 19703399

[67] Xing S, Salinas M, Garcia-Molina A, Höhmann S, Berndtgen R, et al (2013) *SPL8* and miR156-targeted *SPL* genes redundantly regulate Arabidopsis gynoecium differential patterning. Plant J 75: 566–577. 10.1111/tpj.12221 23621152

[68] Zhang H, Zhao X, Li J, Cai H, Deng XW, et al (2014) MicroRNA408 is critical for the *HY5-SPL7* gene network that mediates the coordinated response to light and copper. Plant Cell. 10.1105/tpc.114.127340 PMC4311192 25516599

[69] Manning K, Tör M, Poole M, Hong Y, Thompson AJ, et al (2006) A naturally occurring epigenetic mutation in a gene encoding an SBP-box transcription factor inhibits tomato fruit ripening. Nat Genet 38: 948–952. 10.1038/ng1841 16832354

[70] Miura K, Ikeda M, Matsubara A, Song X-J, Ito M, et al (2010) *Os*SPL14 promotes panicle branching and higher grain productivity in rice. Nat Genet 42: 545–549. 10.1038/ng.592 20495564

[71] Wang L, Cao C, Ma Q, Zeng Q, Wang H, et al (2014) RNA-seq analyses of multiple meristems of soybean: novel and alternative transcripts, evolutionary and functional implications. BMC Plant Biol 14: 169 10.1186/1471-2229-14-169 24939556 PMC4070088

[72] Badel JL, Nomura K, Bandyopadhyay S, Shimizu R, Collmer A, et al (2003) *Pseudomonas syringae* pv. *tomato* DC3000 HopPtoM (CEL ORF3) is important for lesion formation but not growth in tomato and is secreted and translocated by the Hrp type III secretion system in a chaperone-dependent manner. Mol Microbiol 49: 1239–1251. 10.1046/j.1365-2958.2003.03647.x 12940984

[73] Martin K, Kopperud K, Chakrabarty R, Banerjee R, Brooks R, et al (2009) Transient expression in *Nicotiana benthamiana* fluorescent marker lines provides enhanced definition of protein localization, movement and interactions *in planta* . Plant J 59: 150–162. 10.1111/j.1365-313X.2009.03850.x 19309457

[74] Hewezi T, Howe P, Maier TR, Hussey RS, Mitchum MG, et al (2008) Cellulose binding protein from the parasitic nematode *Heterodera schachtii* interacts with Arabidopsis pectin methylesterase: cooperative cell wall modification during parasitism. Plant Cell 20: 3080–3093. 10.1105/tpc.108.063065 19001564 PMC2613657

[75] Waadt R, Schmidt LK, Lohse M, Hashimoto K, Bock R, et al (2008) Multicolor bimolecular fluorescence complementation reveals simultaneous formation of alternative CBL/CIPK complexes *in planta* . Plant J 56: 505–516. 10.1111/j.1365-313X.2008.03612.x 18643980

[76] Keogh RC, Deverall BJ, McLeod S (1980) Comparison of histological and physiological responses to *Phakopsora pachyrhizi* in resistant and susceptible soybean. Trans Br Mycol Soc 74: 329–333. 10.1016/S0007-1536(80)80163-X

[77] Adam L, Somerville SC (1996) Genetic characterization of five powdery mildew disease resistance loci in *Arabidopsis thaliana* . Plant J 9: 341–356. 10.1046/j.1365-313X.1996.09030341.x 8919911

[78] Becker DM, Lundblad V (2001) Introduction of DNA into yeast cells. Curr Protoc Mol Biol Chapter 13: Unit13.7. 10.1002/0471142727.mb1307s27 18265102

[79] Treco DA, Lundblad V (2001) Preparation of yeast media. Curr Protoc Mol Biol Chapter 13: Unit13.1. 10.1002/0471142727.mb1301s23 18265093

[80] An G, Ebert PR, Mitra A, Ha SB (1988) Binary vectors in “Plant Molecular Biology Manual.” Gelvin SB, Schilperoort RA, Verma DPS, editors Dordrecht: Springer Netherlands A3: 1–19 p. 10.1007/978-94-009-0951-9

[81] Vieira A, Talhinhas P, Loureiro A, Duplessis S, Fernandez D, et al (2011) Validation of RT-qPCR reference genes for in planta expression studies in *Hemileia vastatrix*, the causal agent of coffee leaf rust. Fungal Biol 115: 891–901. 10.1016/j.funbio.2011.07.002 21872186

[82] Hu R, Fan C, Li H, Zhang Q, Fu Y-F (2009) Evaluation of putative reference genes for gene expression normalization in soybean by quantitative real-time RT-PCR. BMC Mol Biol 10: 93 10.1186/1471-2199-10-93 19785741 PMC2761916

[83] Liu J-Z, Horstman HD, Braun E, Graham MA, Zhang C, et al (2011) Soybean homologs of MPK4 negatively regulate defense responses and positively regulate growth and development. Plant Physiol 157: 1363–1378. 10.1104/pp.111.185686 21878550 PMC3252160

