# Supplementary figures and images for: A Small Cysteine-Rich Protein from the Asian Soybean Rust Fungus, Phakopsora pachyrhizi, Suppresses Plant Immunity
Source: PLoS Pathog. 2016 Sep 27;12(9):e1005827. doi: 10.1371/journal.ppat.1005827 (PMC5038961; doi:10.1371/journal.ppat.1005827)

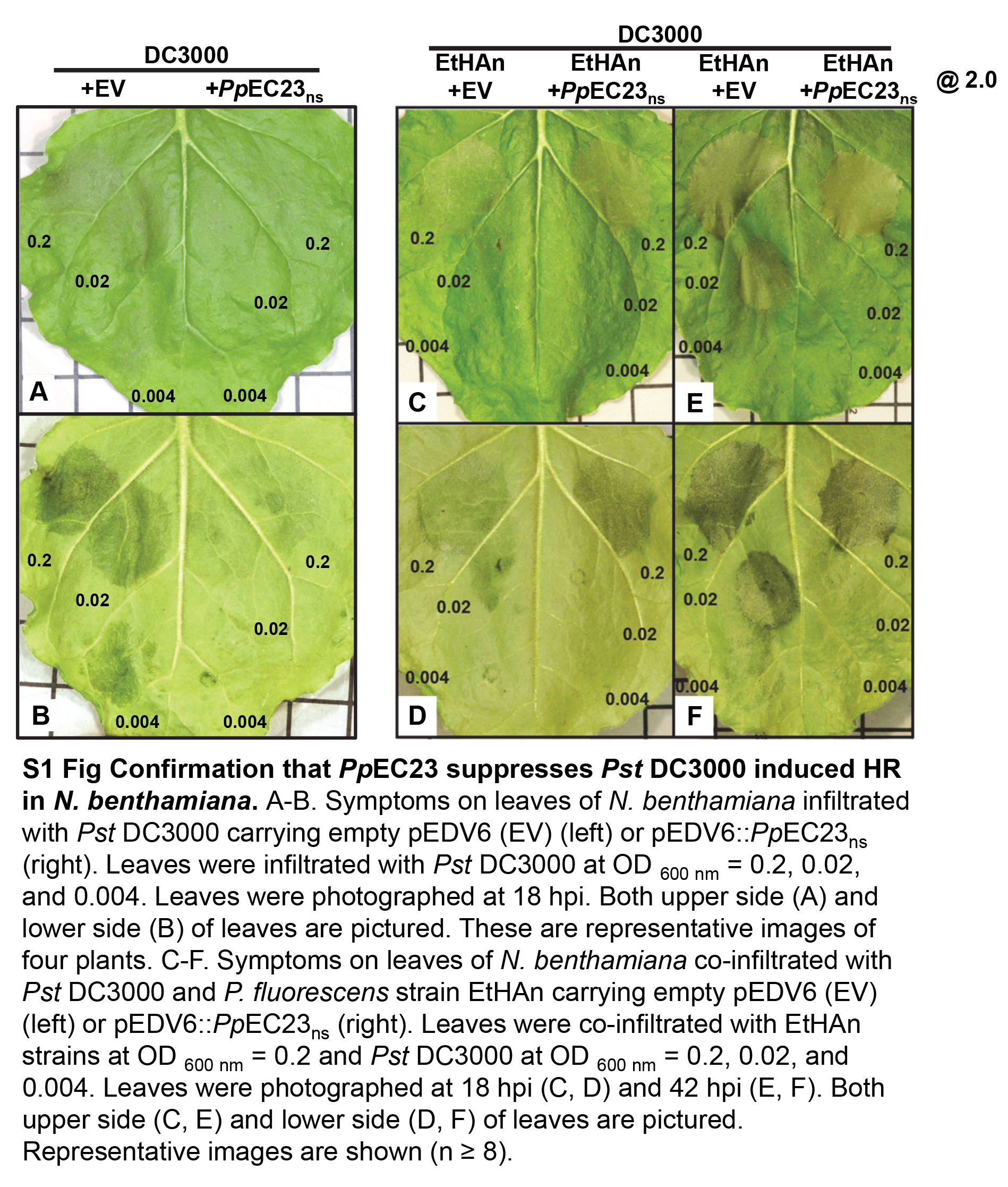

Supplement: S1 Fig — (TIF) [file ppat.1005827.s001.tif]

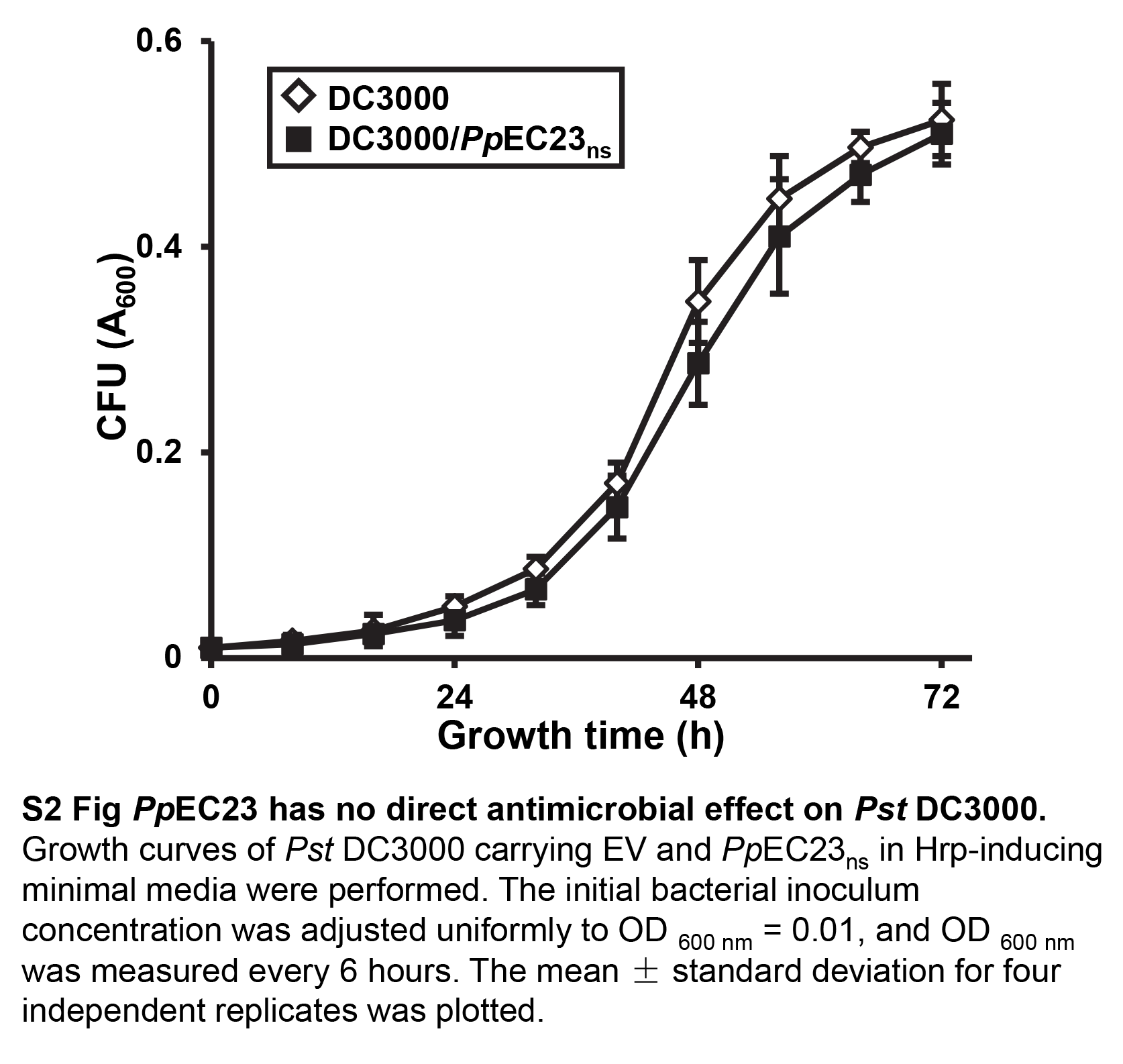

Supplement: S2 Fig — (TIF) [file ppat.1005827.s002.tif]

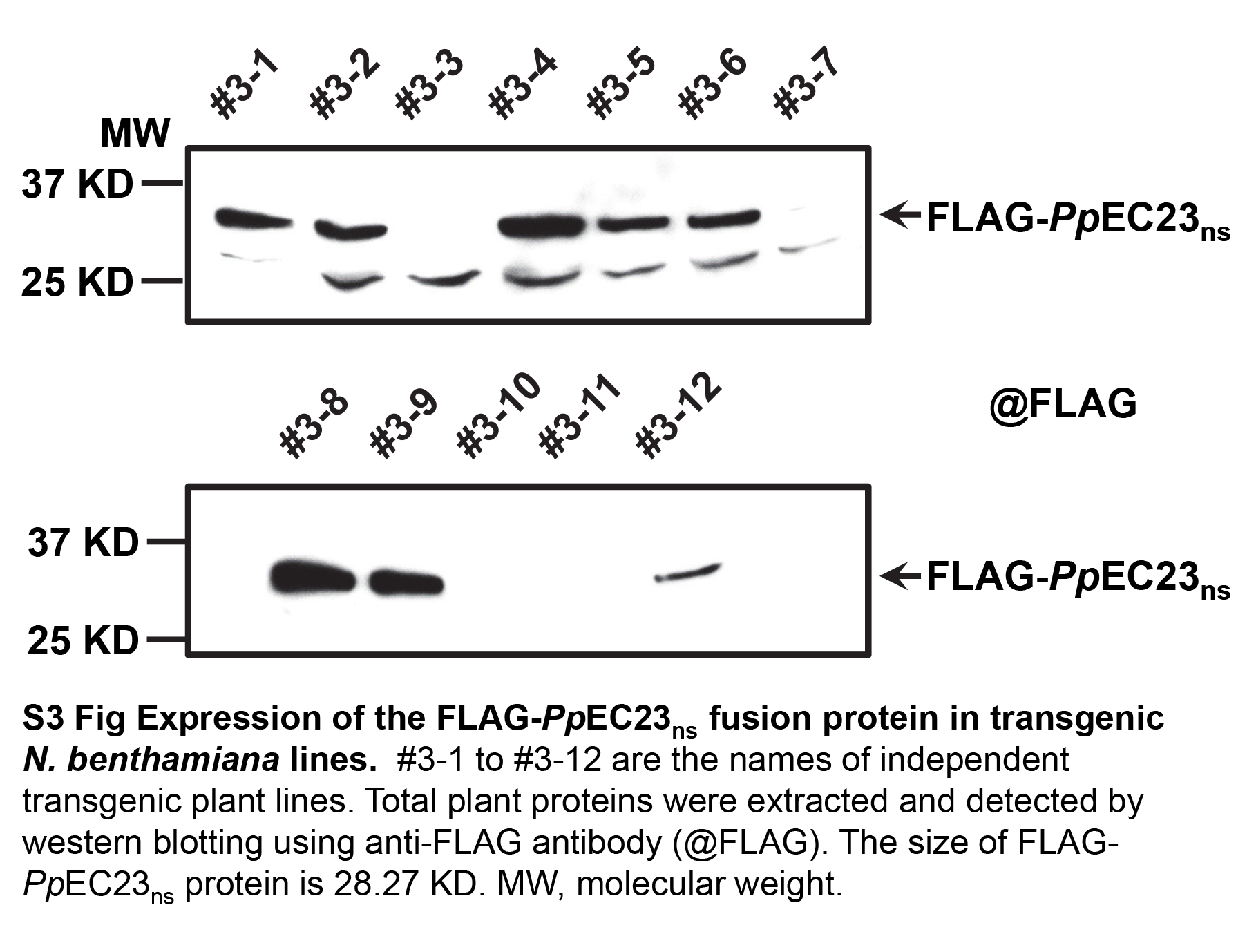

Supplement: S3 Fig — (TIF) [file ppat.1005827.s003.tif]

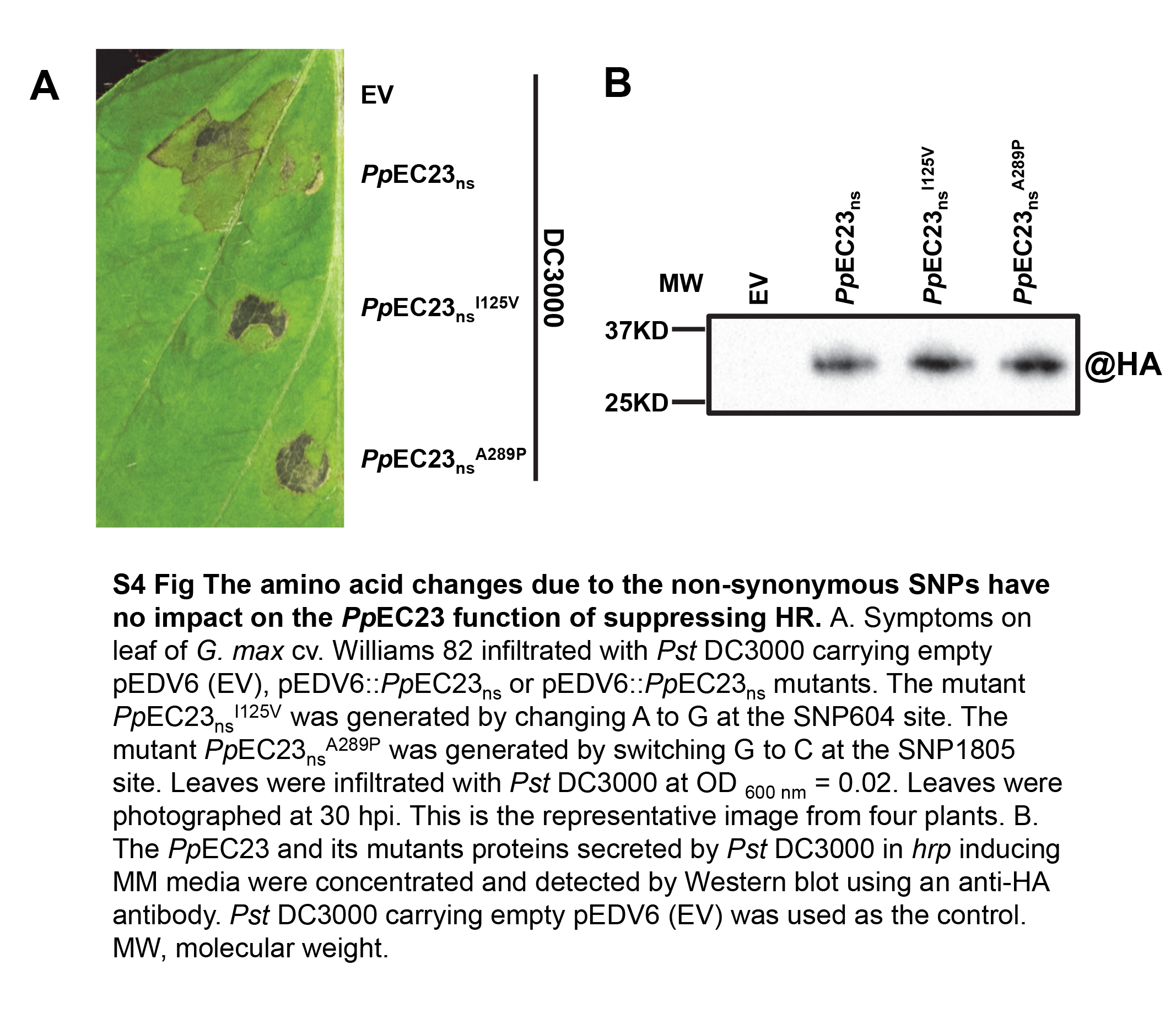

Supplement: S4 Fig — (TIF) [file ppat.1005827.s004.tif]

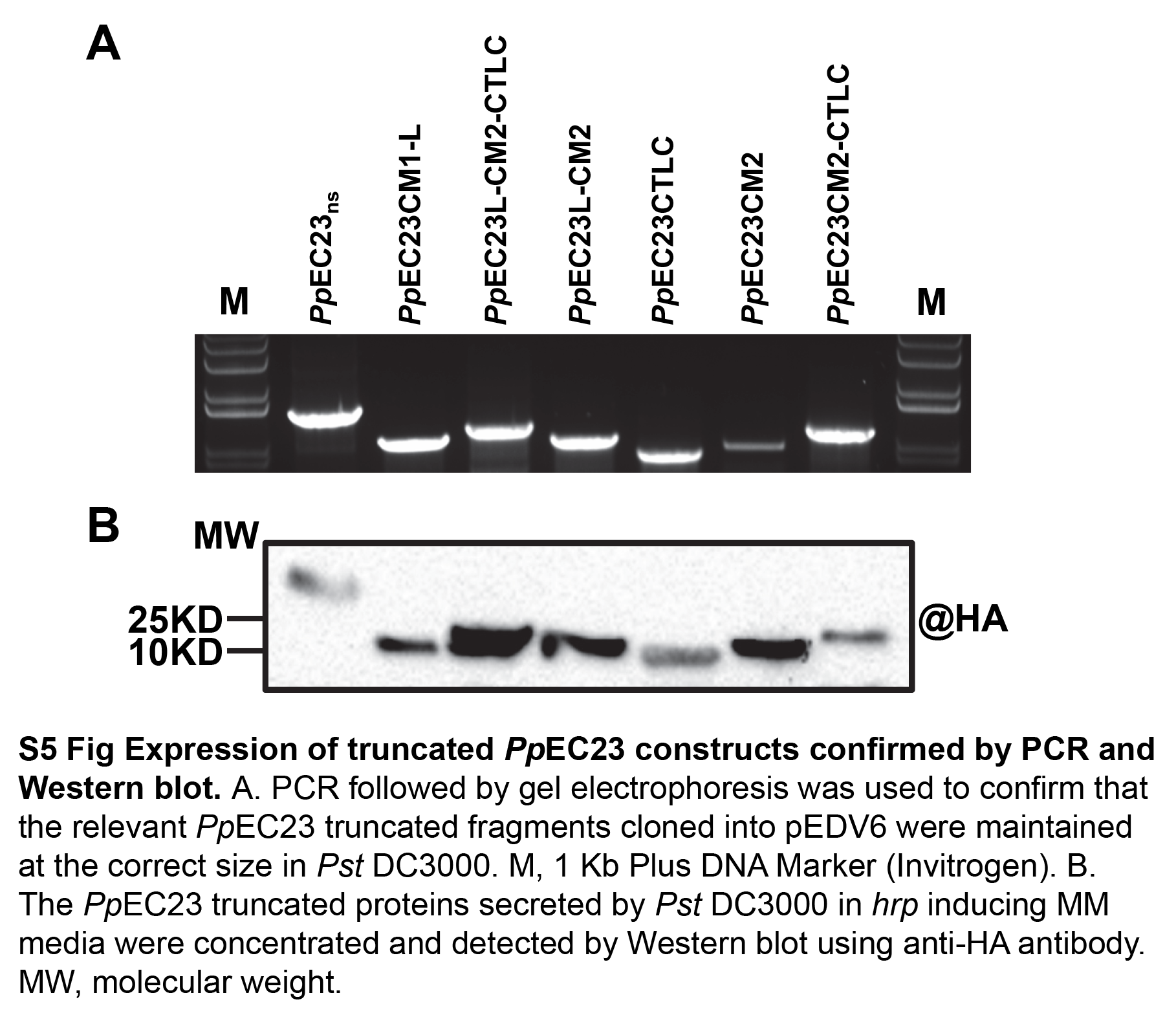

Supplement: S5 Fig — (TIF) [file ppat.1005827.s005.tif]

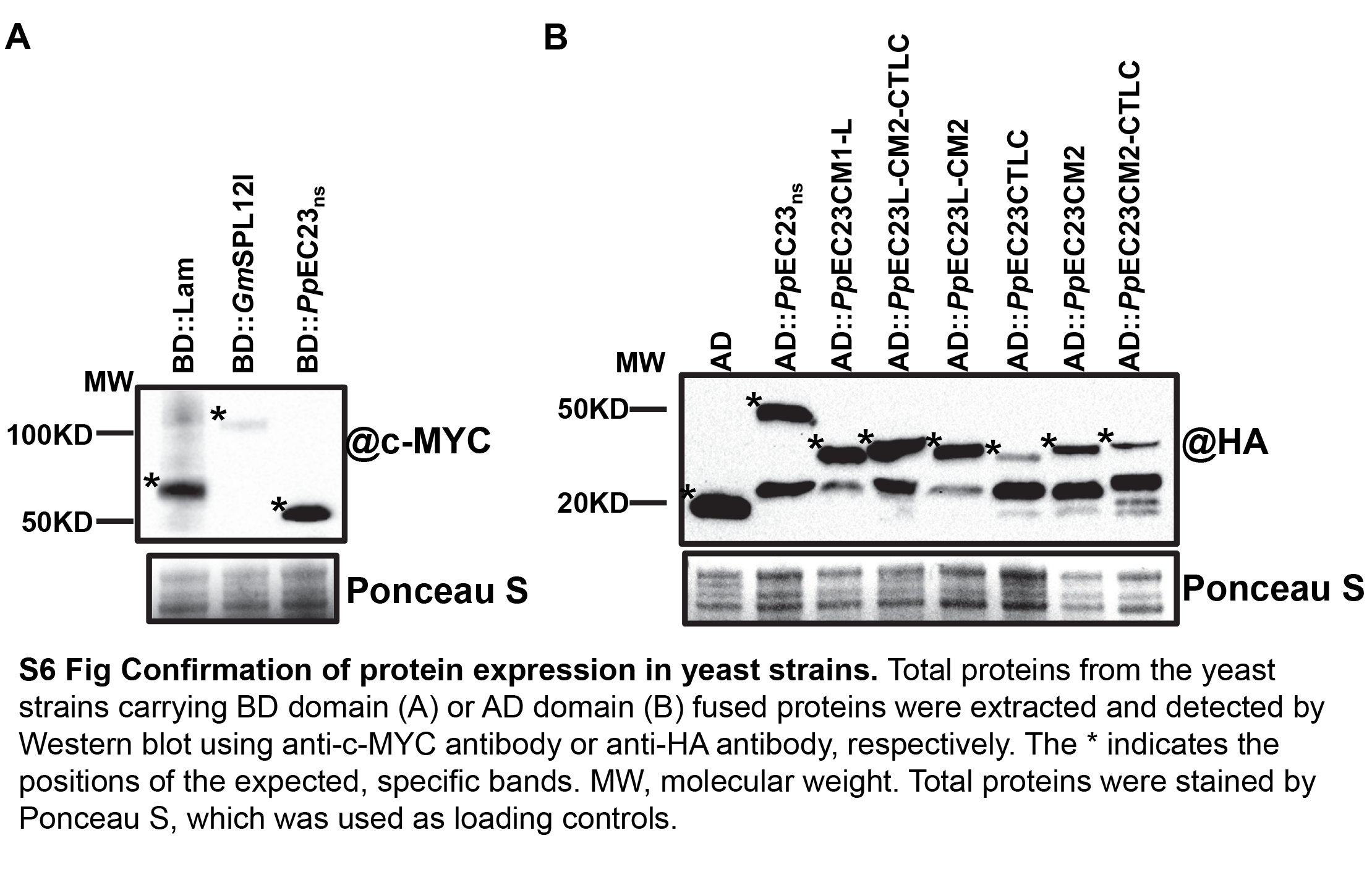

Supplement: S6 Fig — (TIF) [file ppat.1005827.s006.tif]

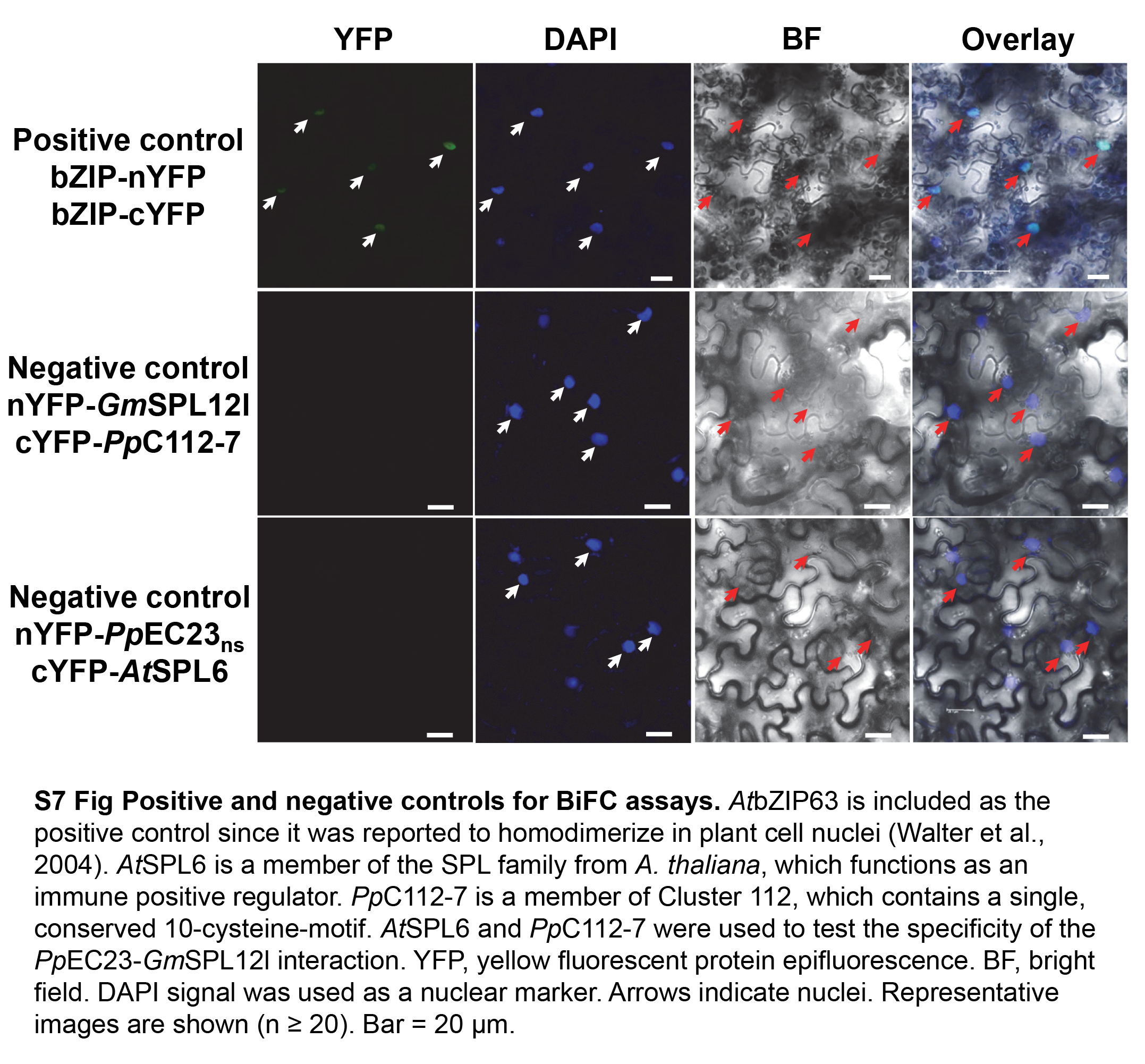

Supplement: S7 Fig — (TIF) [file ppat.1005827.s007.tif]

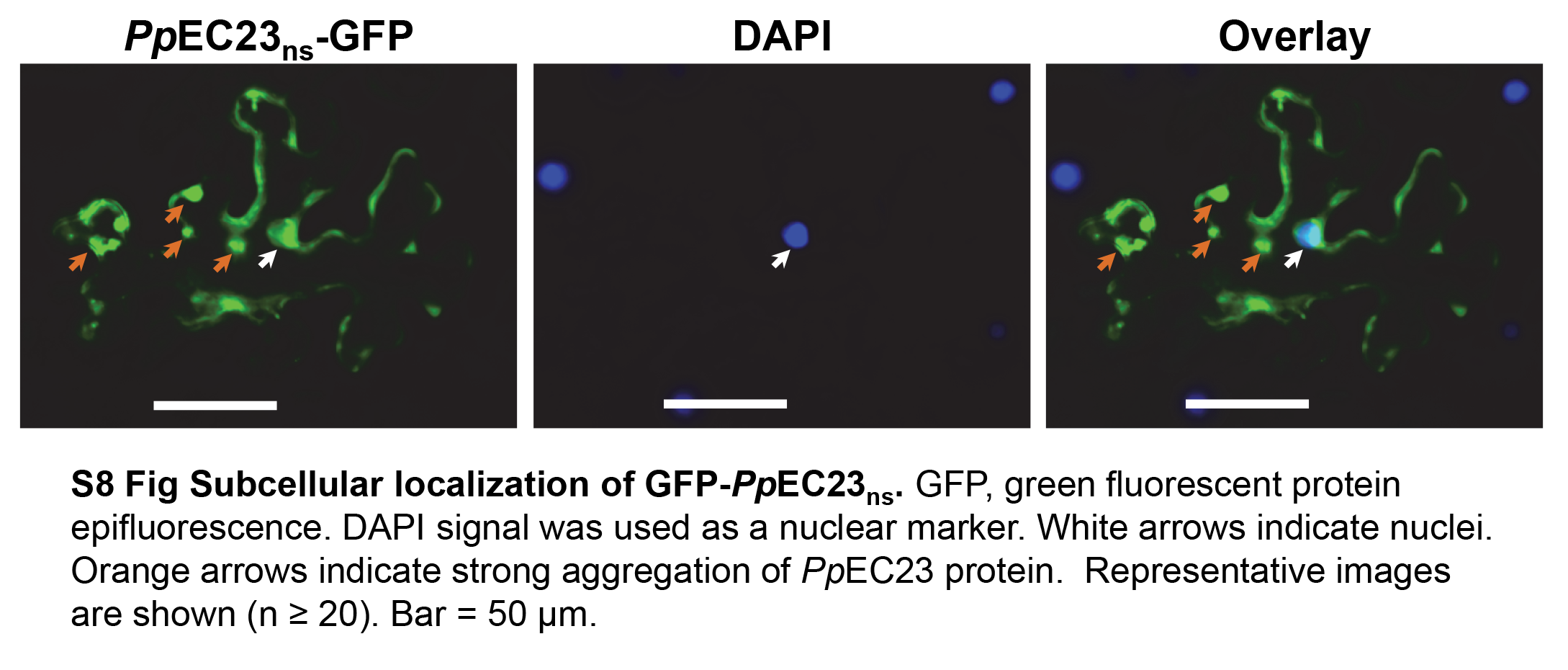

Supplement: S8 Fig — (TIF) [file ppat.1005827.s008.tif]

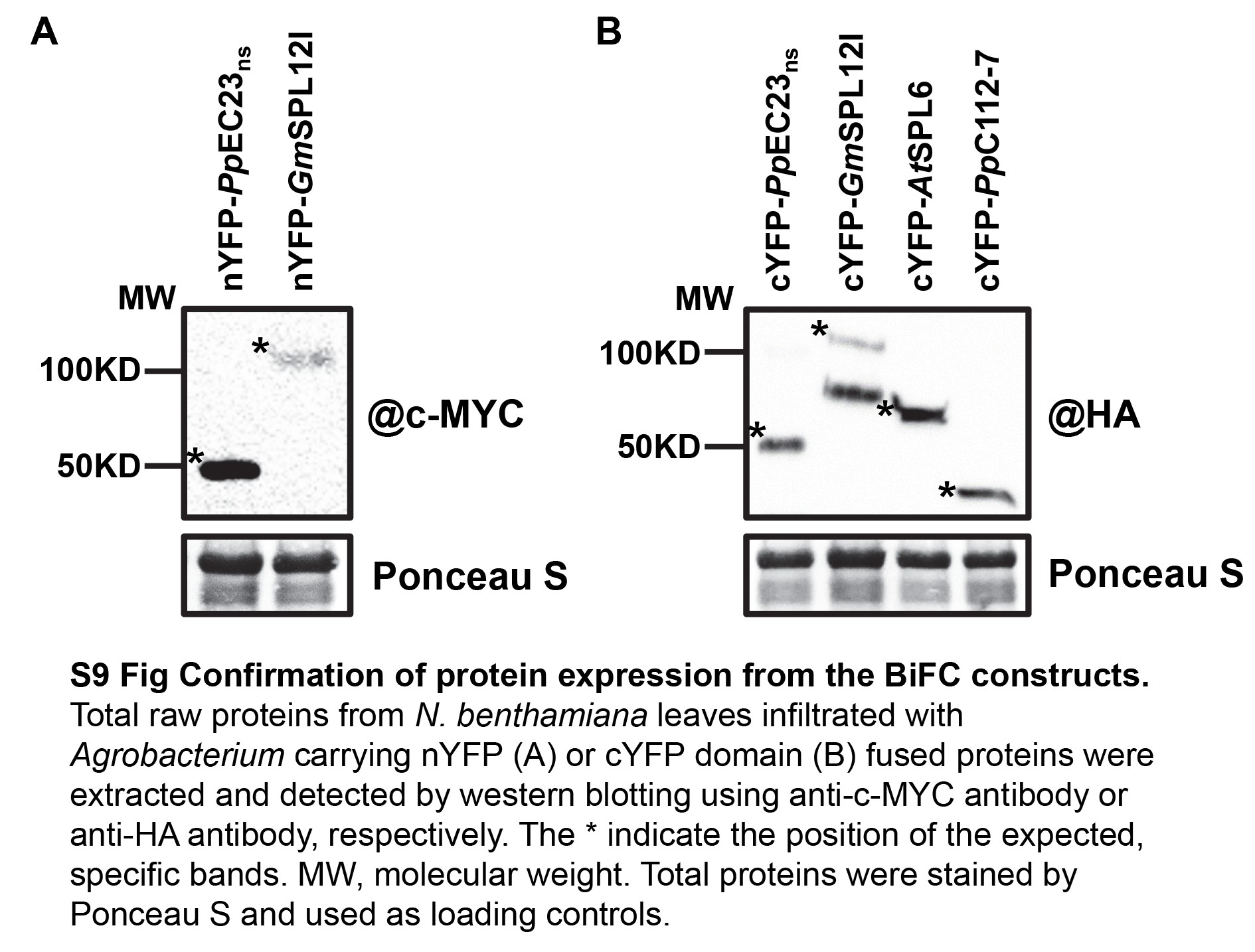

Supplement: S9 Fig — (TIF) [file ppat.1005827.s009.tif]

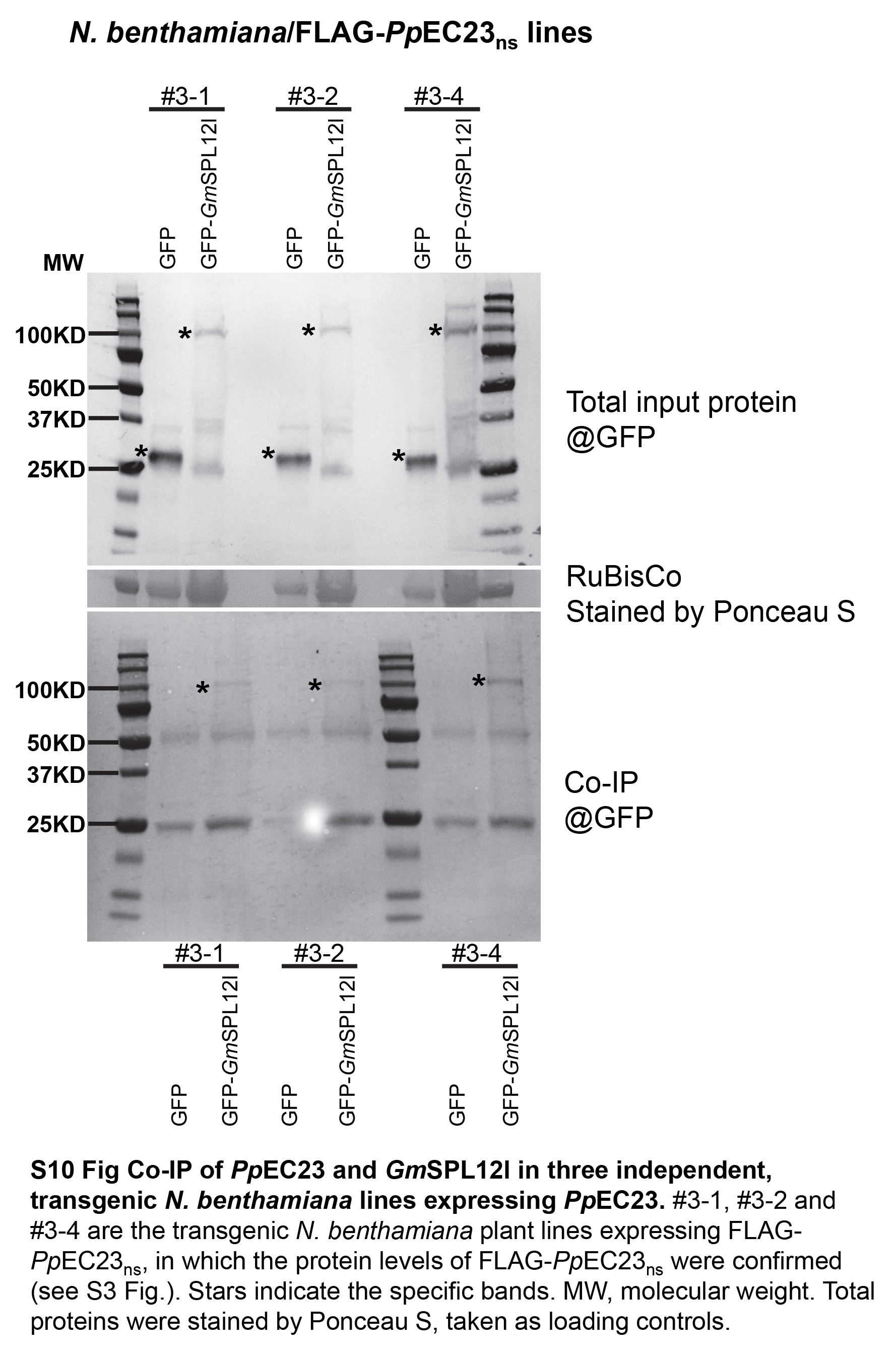

Supplement: S10 Fig — (TIF) [file ppat.1005827.s010.tif]

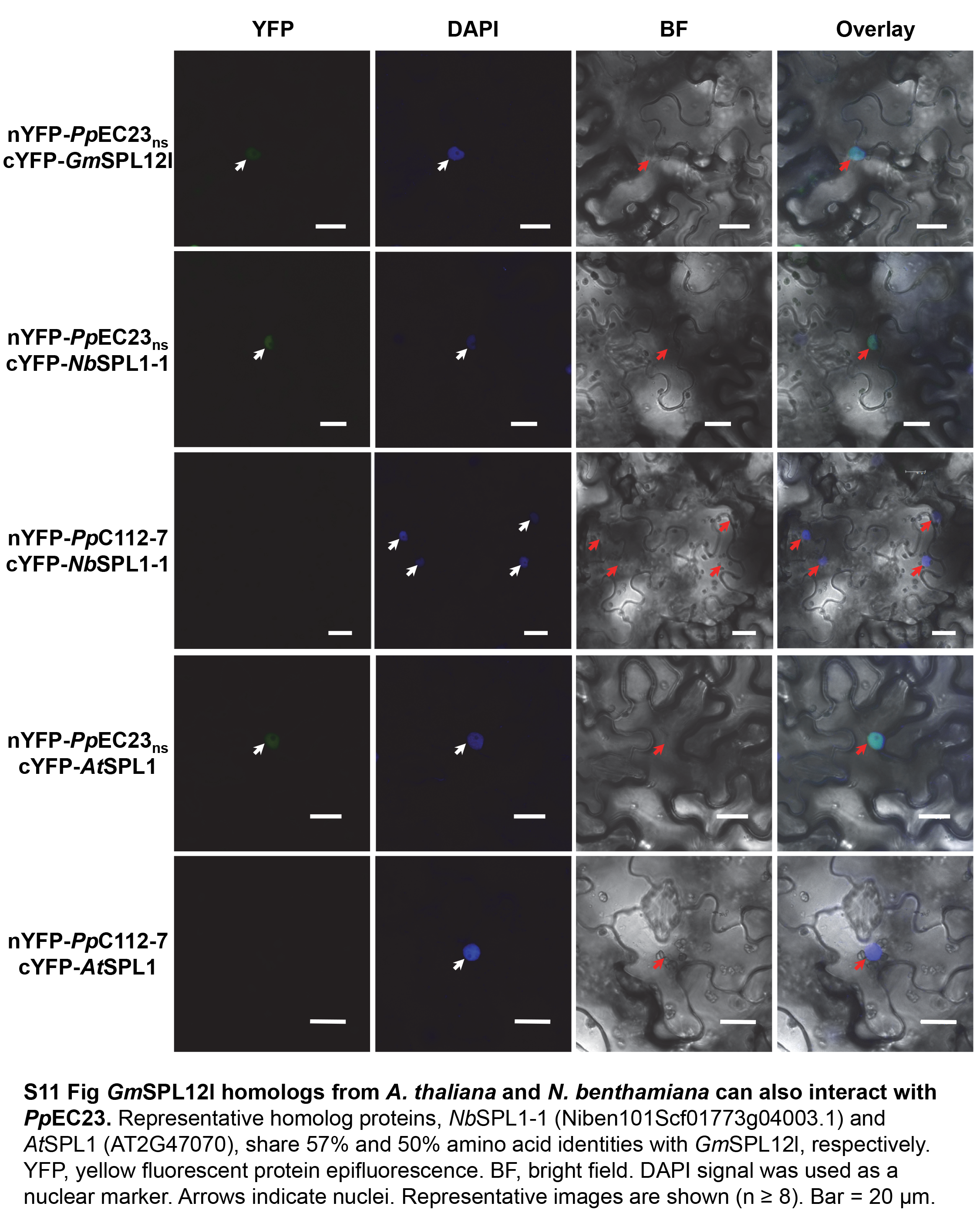

Supplement: S11 Fig — (TIF) [file ppat.1005827.s011.tif]

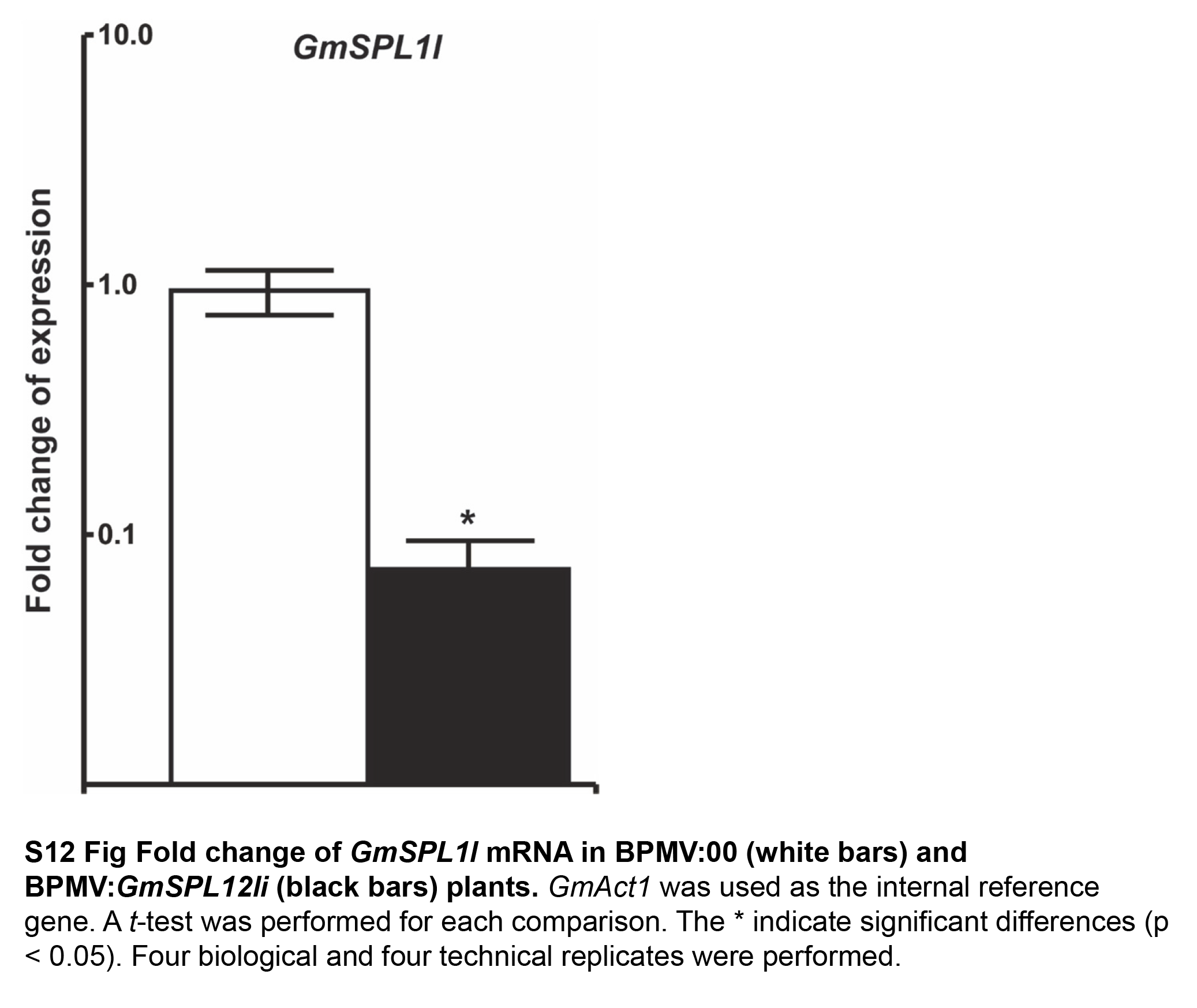

Supplement: S12 Fig — (TIF) [file ppat.1005827.s012.tif]

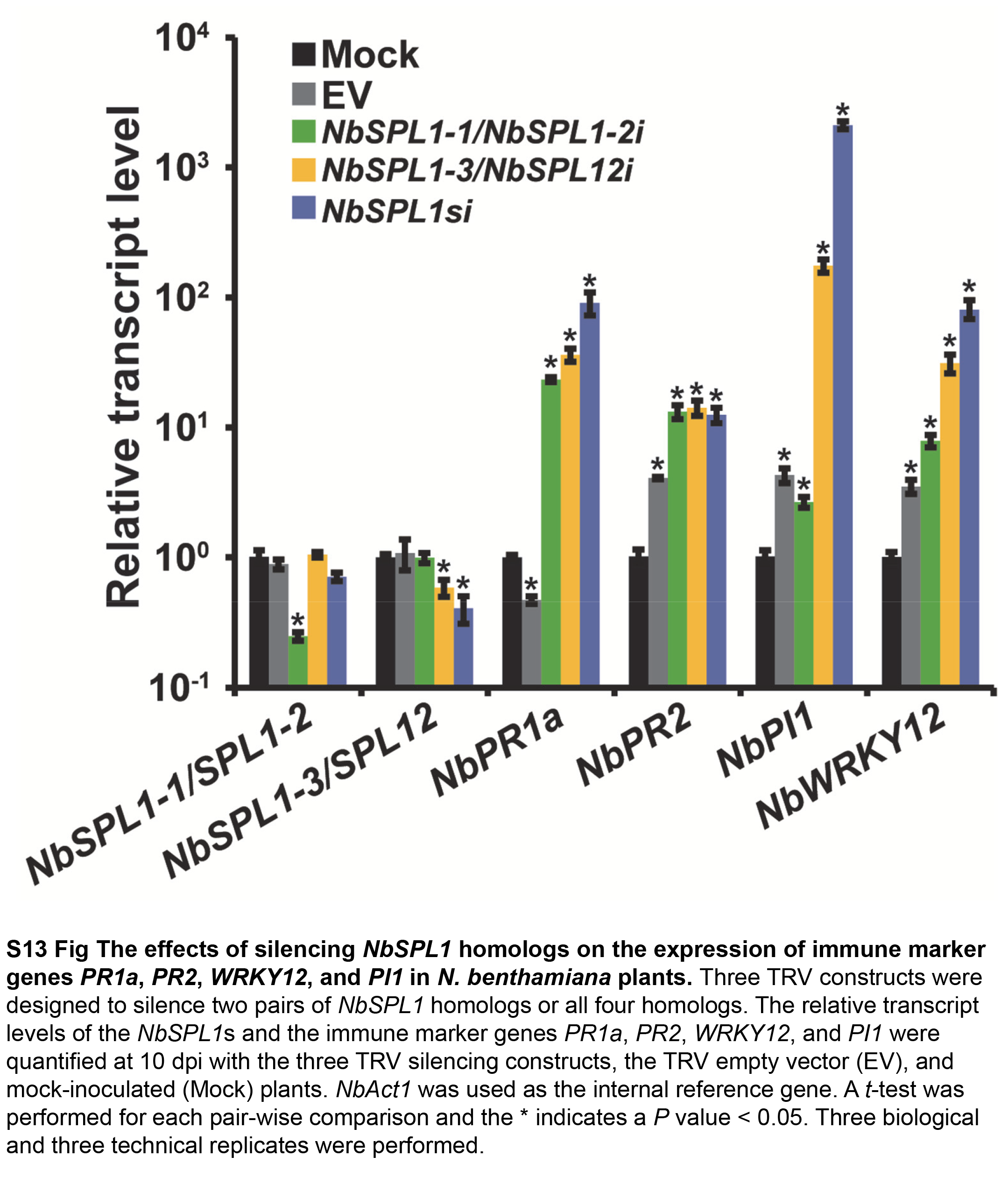

Supplement: S13 Fig — (TIF) [file ppat.1005827.s013.tif]
