# Supplementary material for: A Small Cysteine-Rich Protein from the Asian Soybean Rust Fungus, Phakopsora pachyrhizi, Suppresses Plant Immunity
Source: PLoS Pathog. 2016 Sep 27;12(9):e1005827. doi: 10.1371/journal.ppat.1005827 (PMC5038961; doi:10.1371/journal.ppat.1005827)
Supplement: S1 Table — (DOCX) [file ppat.1005827.s014.docx]

**S1 Table. Primers used in this study.**

| Primer pairs | Purposes |
| --- | --- |
| F: TCGGAGCCAAGCGGA  R: tcaGAGCACGGCGACACCA | To construct pCR8-*Pp*EC23_ns_ for gateway entry clone |
| F: TCGGAGCCAAGCGGA  R: tcaTTCTTTTGAAGTCGATCCGTTGT | To construct pCR8-*Pp*EC23CM1-L for gateway entry clone |
| F: CCTCCAACAGGTTCCAATCAA  R: tcaGAGCACGGCGACACCA | To construct pCR8-*Pp*EC23L-CM2-CTLC for gateway entry clone |
| F: CCTCCAACAGGTTCCAATCAA  R: tcaATCGGTACATCCAGAGCA | To construct pCR8-*Pp*EC23L-CM2 for gateway entry clone |
| F: CAATCATCATCTGGTTCCTCT  R: tcaGAGCACGGCGACACCA | To construct pCR8-*Pp*EC23CTLC for gateway entry clone |
| F: GGCCAAACTCTACAGTGCAC  R: tcaATCGGTACATCCAGAGCA | To construct pCR8-*Pp*EC23CM2 for gateway entry clone |
| F: GGCCAAACTCTACAGTGCAC  R: tcaGAGCACGGCGACACCA | To construct pCR8-*Pp*EC23CM2-CTLC for gateway entry clone |
| F:CGG CCA TTA CGG CCA TGT ATC ACT TAA CCT TTG TCC  R:GAG GGC CGA GGC GGC CAG CACGGCGACACCAATC | To construct pSuc2t7M13ori::*Pp*EC23 for yeast signal trap assay |
| F:CGG CCA TTA CGG CCATGGCTTCTACTTCGGAGCCAAG  R:GAG GGC CGA GGC GGC CAG CAC GGC GAC ACC AAT C | To construct pSuc2t7M13ori::*Pp*EC23_ns_ for yeast signal trap assay |
| F:CGG CCA TTA CGG CCA TGT ATC ACT TAA CCT TTG TCC  R:GAG GGC CGA GGC GGC CGTCGATCCGTTGTTTCCTGG | To construct pSuc2t7M13ori::*Pp*EC23Sp-CM1-L for yeast signal trap assay |
| F:CGG CCA TTA CGG CCA TGCCAGGAAACAACGGATCGAC  R:GAG GGC CGA GGC GGC CAG CAC GGC GAC ACC AAT C | To construct pSuc2t7M13ori::*Pp*EC23L-CM2-CTLC for yeast signal trap assay |
| F:CGG CCA TTA CGG CCA TGT ATC ACT TAA CCT TTG TCC  R:GAG GGC CGA GGC GGC CGCTCCGTTAGCGGACAAAAA | To construct pSuc2t7M13ori::*Pp*EC23Sp for yeast signal trap assay |
| F: CGCGG ATC Cct TCGGAGCCAAGCGGA  R: CCG CTC GAG GAGCACGGCGACACCA | To construct pLexA*-Pp*EC23_ns_ for Y2H library screening |
| F: GGAATTCCATATG TCGGAGCCAAGCGGA  R: CCGCTCGAG tcaGAGCACGGCGACACCA | To construct pGADT7*-Pp*EC23_ns_ for Y2H confirmation |
| F: CATATG TCGGAGCCAAGCGGA  R: CCG CTC GAG tcaTTCTTTTGAAGTCGATCCGTTGT | To construct pGADT7-*Pp*EC23CM1-L for Y2H confirmation |
| F: CATATG CCTCCAACAGGTTCCAATCAA  R: CCGCTCGAG tcaGAGCACGGCGACACCA | To construct pGADT7-*Pp*EC23L-CM2-CTLC for Y2H confirmation |
| F: CATATG CCTCCAACAGGTTCCAATCAA  R: CTCGAG tcaATCGGTACATCCAGAGCA | To construct pGADT7-*Pp*EC23L-CM2 for Y2H confirmation |
| F: CATATG CAATCATCATCTGGTTCCTCT  R: CCGCTCGAG tcaGAGCACGGCGACACCA | To construct pGADT7-*Pp*EC23CTLC for Y2H confirmation |
| F: CATATG GGCCAAACTCTACAGTGCAC  R: CTCGAG tcaATCGGTACATCCAGAGCA | To construct pGADT7-*Pp*EC23CM2 for Y2H confirmation |
| F: CATATG GGCCAAACTCTACAGTGCAC  R: CCGCTCGAG tcaGAGCACGGCGACACCA | To construct pGADT7-*Pp*EC23CM2-CTLC for Y2H confirmation |
| F: GGAATTCCATATG TCGGAGCCAAGCGGA  R: ACGCGTCGAC TCA GAG CAC GGC GAC ACC | To construct pGBKT7*-Pp*EC23_ns_ for Y2H confirmation |
| F: CGCGGATCC atgTCGGAGCCAAGCGGA  R: CCGCTCGAG tcaGAGCACGGCGACACCA | To construct phygII-SPYNE(R)155*-Pp*EC23_ns_ for BiFC assay |
| F: CGCGGATCC atgTCGGAGCCAAGCGGA  R: CCGCTCGAG tcaGAGCACGGCGACACCA | To construct pkanII-VYCE(R)*-Pp*EC23_ns_ for BiFC assay |
| F: CGCGGATCC ATG GAC TAC AAA GAC GAT GAC  R: ACGCGTCGAC TCA GAG CAC GGC GAC ACC | To construct pBI121-FLAG*-Pp*EC23_ns_ for *A. thaliana* transformation * |
| F: CCGGAATTC ATG GAA GCT AGA TTT GGA GCT GA  R: ACGCGTCGAC TTAGCTTGTTCCAAAGTCCAAATTCT | To construct pGBKT7-*Gm*SPL12l for Y2H confirmation |
| F: ATG GAA GCT AGA TTT GGA GCT GA  R: TTAGCTTGTTCCAAAGTCCAAATTCT | To construct pCR8-*Gm*SPL12l for gateway entry clone |
| F: CGGACTAGT ATG GAA GCT AGA TTT GGA GCT GA  R: CCGCTCGAG TTAGCTTGTTCCAAAGTCCAAATTCT | To construct phygII-SPYNE(R)155-*Gm*SPL12l for BiFC assay |
| F: CGGACTAGT ATG GAA GCT AGA TTT GGA GCT GA  R: CCGCTCGAG TTAGCTTGTTCCAAAGTCCAAATTCT | To construct pkanII-VYCE(R)- *Gm*SPL12l for BiFC assay |
| F: CGCGGATCCAGAAATATAAAACTTTCCTCAAGAATTGAT  R: GGGGTACC TTA TGC ATC AAC ACA GCC TTC | To construct pkanII-VYCE(R)-*Pp*C112-7 for BiFC assay |
| F: CGCGGATCC ATGGATTCTTGGAGCTACGG  R: GGGGTACC TTA GGA GCC AGG GAA ATA AAG C | To construct pkanII-VYCE(R)-*At*SPL6 for BiFC assay |
| F: aaactcgagGCTCATGTGGTGGTTGAGATT  R: aaaggatccCACCTGAAGGGTCGGAAC | To construct pBPMV-IA-V2-*Gm*SPL12l for Soybean gene silencing |
| F: agttgctcgagtgactggtg  R: catcttgggcagccaacatg | qRT-PCR for *RPS14* (*Pp*_contig00153) |
| F: acc tcc cgt tca gct agt ct  R: aat tca tca gag tcg gcc cc | qRT-PCR for *PDK* (*Pp*_contig02726) |
| F: gcc tct gga tac ctg ctc aag  R: acc tcc tcc tca aac tcc tct g | qRT-PCR for *Ukn2* (Glyma06g04180.1) |
| F: cctaagaggtttgagttagctg  R: ctgcaaagatgatttgcctctc | qRT-PCR for *PpEC23* |
| F: GAGCTATGAATTGCCTGATGG  R:CGTTTCATGAATTCCAGTAGC | qRT-PCR for *GmAct* |
| F: AGAGGCAGAGGTGGGTTCT  R: TCACCAACAAAGTTGCCAGG | qRT-PCR for *GmPR1a* |
| F: GCAGTTTTTCCCCACCGGG  R: TCTTGTCCCCTTCCTTGTTCC | qRT-PCR for *GmSPL12l* |
| F: gaa gct cag agt cgc aca ga  R: agg ccg tat gta gct ctc ca | qRT-PCR for *GmSPL1l* |
| KP680: ATGTATCACTTAACCTTTGTCCTCTTT  KP713: CTAACTCAAACCTCTTAGG | Amplify *PpEC23* genomic sequence |
| F: GTTGCTATACAAGCTGTTCTCTCG  R: GTCAAGACGAAGAATGACATGTGG | qRT-PCR for *NbAct* |
| F: CGACCAGGTAGCAGCCTATG  R: TCTCAACAGCCTTAGCAGCC | qRT-PCR for *NbPR1a* |
| F: GGGCTGTTAATTTGCAGTATCC  R: GGTTTATAACATCTTGGTCTGATGG | qRT-PCR for *NbPR2* |
| F: CTCATCAGCTAGTTCATTTGATGC  R: AGCTCGGTCTTTGTTCTAAAAGC | qRT-PCR for *NbWRKY12* |
| F: CTTCAAAGACTATGGTGAAGTTTGC  R: CAGACTGAGACACATCAAGTTGC | qRT-PCR for *NbPI1* |
| F: TGGTTGATATTCCTATTGTTCCATCTATT  R: CGA GAA ATG GGT CTT CTC TCG GT | qRT-PCR for *NbSPL1-1/SPL1-2* |
| F: GTTGATATTCCGTATAACCTGTCAGAC  R: TGA ATG AGT CTC CTC TCA GTC TG | qRT-PCR for *NbSPL1-3/SPL12* |
| F: CGGGATCC ATG GAA GCT AGA ATT GAT GAA GG  R: GGGGTACC TCA GCT TGT TCC ATA GTC CAA TA | To construct pkanII-VYCE(R)*-AtSPL1* for BiFC assay |
| F: CGGGATCC ATG GAA GCG AGC GTG  R: GGGGTACC TCA GCT TGT TCC AAA GTC CA | To construct pkanII-VYCE(R)*-NbSPL1-1* for BiFC assay |
| F: GAATTCTTGTTTAGACCTGATTG  R: gaccaatcccTCTTTCTGGTTGCTGTTC | To construct p279-*NbSPL1-1/NbSPL1-2i* |
| F: accagaaagaGGGATTGGTCCTCTTGAG  R: CCAGGGACATCCAAAAGC | To construct p279-*NbSPL1-3/NbSPL12i* |
| F: GAATTCTTGTTTAGACCTGATTG  R: CCAGGGACATCCAAAAGC | To construct p279-*NbSPL1si* |

* : Primers are used for amplifying FLAG-*Pp*EC23_ns_ from pEarleyGate202-*Pp*EC23
